# Extracellular vesicles produced by HIV-1 Nef-expressing cells induce myelin impairment and oligodendrocyte damage in the mouse central nervous system

**DOI:** 10.1186/s12974-024-03124-5

**Published:** 2024-05-13

**Authors:** Jessica K. Schenck, Molly T. Karl, Cheryl Clarkson-Paredes, Ashley Bastin, Tatiana Pushkarsky, Beda Brichacek, Robert H. Miller, Michael I. Bukrinsky

**Affiliations:** School of Medicine and Health Sciences, The George Washington University, 2300 I St NW, Ross Hall 624, Washington, DC 20037 USA

**Keywords:** HIV-1, Nef, Extracellular vesicles, HAND, Myelin, Oligodendrocyte, Astrocyte, Microglia

## Abstract

HIV-associated neurocognitive disorders (HAND) are a spectrum of cognitive impairments that continue to affect approximately half of all HIV-positive individuals despite effective viral suppression through antiretroviral therapy (ART). White matter pathologies have persisted in the ART era, and the degree of white matter damage correlates with the degree of neurocognitive impairment in patients with HAND. The HIV protein Nef has been implicated in HAND pathogenesis, but its effect on white matter damage has not been well characterized. Here, utilizing in vivo, ex vivo, and in vitro methods, we demonstrate that Nef-containing extracellular vesicles (Nef EVs) disrupt myelin sheaths and inflict damage upon oligodendrocytes within the murine central nervous system. Intracranial injection of Nef EVs leads to reduced myelin basic protein (MBP) staining and a decreased number of CC1 + oligodendrocytes in the corpus callosum. Moreover, cerebellar slice cultures treated with Nef EVs exhibit diminished MBP expression and increased presence of unmyelinated axons. Primary mixed brain cultures and enriched oligodendrocyte precursor cell cultures exposed to Nef EVs display a decreased number of O4 + cells, indicative of oligodendrocyte impairment. These findings underscore the potential contribution of Nef EV-mediated damage to oligodendrocytes and myelin maintenance in the pathogenesis of HAND.

**Supplementary Information:**

The online version contains supplementary material available at 10.1186/s12974-024-03124-5.

## Introduction

HIV-associated neurocognitive disorders (HAND) represent a spectrum of cognitive impairments commonly observed in people living with HIV (PLWH), affecting up to half of all HIV-positive individuals. Despite the advent of anti-retroviral therapy (ART), which has substantially reduced the incidence of severe HIV-associated dementia, milder forms of HAND persist even with sustained virologic control [1–6]. Notably, individuals with high CD4 T-cell counts [7–12] and those in the acute and early periods of HIV infection are also at risk of neurocognitive impairment, as damage to the central nervous system (CNS) occurs shortly after viral infection [13].

In the era of ART, the clinical manifestations of HIV-associated neuropathology have shifted away from severe acute disease defined by encephalopathy and neurodegeneration towards more subtle neuroinflammatory changes and sublethal alterations in neuronal functions [14], with white matter abnormalities persisting. Studies utilizing neuroimaging techniques have identified white matter damage, including increased white matter hyperintensity and corpus callosum thinning, in HIV-infected individuals on ART [15–18]. Transcriptome analyses of patients with HAND on ART have revealed downregulation of genes associated with oligodendrocyte maturation and myelin metabolism [19, 20]. Importantly, the severity of white matter damage correlates with the degree of neurocognitive impairment in patients with HAND [21–25], though the mechanisms underlying HIV-associated white matter damage remain incompletely understood.

Oligodendrocyte injury is a hallmark of white matter damage and demyelination, processes demonstrated in both cell culture and animal models of HIV-associated neuropathology [26, 27]. Oligodendrocyte-specific expression of Nef was shown to affect appropriate differentiation of oligodendrocytes in vitro [28]. However, HIV does not directly infect oligodendrocytes; microglia and astrocytes are recognized HIV reservoirs within the CNS and can produce viral proteins, including Nef, even during successful ART [29–31]. The release of these viral proteins via extracellular vesicles may mediate the “bystander” effects of HIV infection on uninfected cells. The HIV protein Nef is responsible for many pathogenic effects of HIV infection [32] and has been implicated as a key player in HAND pathogenesis [33–35]. Recent animal studies have shown that astrocytic expression of Nef induces deficits in spatial and recognition memory, which is correlated with increased CNS infiltration of peripheral macrophages and neuroinflammation [36, 37]. Astrocytes are known to be essential for the development and maintenance of oligodendrocytes, and Nef-mediated white matter damage likely involves perturbation of astrocyte–oligodendrocyte–microglia crosstalk, which is important for myelin formation and maintenance, and aberrant astrocyte or microglial function has major consequences for oligodendrocyte physiology and white matter integrity [38, 39].

Previous research has demonstrated that Nef impairs cellular cholesterol homeostasis in macrophages by decreasing the abundance and functional activity of the critical cholesterol transporter ATP-binding cassette transporter A1 (ABCA1) and inhibiting cholesterol efflux [40]. This ultimately alters the lipid composition of the macrophage cellular membrane, potentiating inflammatory responses [41]. Importantly, Nef-containing extracellular vesicles (Nef EVs) impair ABCA1-mediated cholesterol efflux in uninfected cells [42], similar to the effect of Nef in HIV-infected cells. This underscores the potential significance of Nef and Nef EVs in HAND pathogenesis in ART-treated patients, as Nef can be detected in the plasma of aviremic individuals [43], indicative of its production by latent HIV reservoirs despite effective antiretroviral therapy. Additionally, the presence of Nef EVs in the brains of ART-treated PLWH may predict neurocognitive impairment status [44].

The intricate relationship between cholesterol metabolism and myelin synthesis underscores the importance of understanding how Nef-mediated disruption in cholesterol homeostasis may contribute to HAND. Oligodendrocytes, responsible for myelin formation, rely heavily on cholesterol for the maintenance of myelin sheaths, the fatty insulation surrounding axons [45]. In the CNS, where the blood‒brain barrier restricts the entry of LDL particles, cholesterol homeostasis depends on recycling and de novo biosynthesis [46]. While oligodendrocytes contribute to the synthesis of cholesterol incorporated into myelin membranes during development [47, 48], astrocytes primarily produce cholesterol in the adult CNS [47–49]. Furthermore, cholesterol recycling by phagocytic astrocytes and microglia is crucial for myelin repair and overall cholesterol homeostasis. ABCA1, present in both astrocytes and mature oligodendrocytes, plays a pivotal role in myelination by facilitating cholesterol transport [50]. However, the function of ABCA1-mediated cholesterol transport may be compromised by Nef-containing EVs, thereby disrupting myelin formation or maintenance by oligodendrocytes. In this study, we employed multiple methodologies to explore the impact of Nef EVs on myelin sheaths, aiming to elucidate a novel mechanism underlying CNS demyelination in HAND. We hypothesize that Nef-induced downregulation and inactivation of ABCA1 disrupt the intricate process of myelin formation or maintenance by oligodendrocytes. This proposed mechanism may provide insights into why mild forms of cognitive impairment persist despite undetectable viral loads in ART-treated HIV patients.

## Methods

### Animals

C57BL/6J mice (Jackson Laboratory) were maintained under standard mouse husbandry conditions, including a 12:12 h light:dark cycle and ad libitum access to food and water. All the experimental procedures were approved by the George Washington University Institutional Animal Care and Use Committee.

### Isolation and purification of extracellular vesicles (EVs)

EVs were produced as previously described [51]. Briefly, HEK293T cells were transfected with either a pcDNA3.1 vector expressing Nef from HIV-1 NL4-3 to produce Nef EVs or an empty vector to produce control (Ctrl) EVs; the cells were subsequently grown in DMEM supplemented with 10% heat-inactivated exosome-depleted fetal bovine serum (FBS) (Thermo Fisher, A2720801). At 48 h post transfection, the medium was collected, and the EVs were isolated via differential centrifugation [52]. First, culture supernatants were pre-clarified to remove cells and cellular debris by centrifugation at 500×*g* for 10 min at 4 °C and subsequently clarified to remove any remaining debris and large apoptotic bodies by centrifugation at 3000×*g* for 30 min at 4 °C. Nef and Ctrl EVs were pelleted via a final centrifugation step at 100,000×*g* for 75 min at 4 °C. The pellet was resuspended in sterile saline supplemented with 10% mouse serum (Innovative Research, IGMSBCSER) for in vivo brain injections or in DMEM/F-12 (Thermo Fisher, 11320033) supplemented with 1% FBS for in vitro and ex vivo culture, aliquoted, and frozen at − 70 °C. The utilization of frozen samples was indispensable to ensure consistency of Nef concentrations in the EV preparations utilized across various experiments. This approach also facilitated the validation of the biological activity of EV preparations in vitro before undertaking more complex in vivo experimental procedures. Recent studies have shown that freezing of EVs leads to particle loss and fusion [53, 54]. However, considering the primary focus of our study on Nef, we ensured consistent Nef concentrations as a priority. Additionally, since we consistently compared Nef EVs to control EVs prepared concurrently, any potential damage incurred during freezing was anticipated to affect both types of EVs equally, thus not influencing the outcome of comparisons. Ultimately, any potential damage to EVs could only attenuate the observed effects, rather than alter the conclusions drawn from this study. To ensure consistency, we aliquoted the EVs, froze them, and utilized the aliquots in all in vitro and in vivo experiments, thereby avoiding repeated freeze–thaw cycles or the use of EV batches with varying Nef concentrations. The concentrations of the Nef and Ctrl EVs were estimated using Tunable Resistive Pulse Sensing technology on the Exoid instrument (Izon Sciences Ltd) (Supplemental Fig. S1A). Specifically, 3.2 × 10^9^ particles in 10 µL were used to treat ex vivo slice cultures and 1 × 10^5^ cells in vitro*,* while 4.3 × 10^9^ particles in 5 µL were injected into mouse brains in vivo. Nef EVs carried 0.67 ng Nef per 4.4 × 10^10^ EV particles, as measured by a home-made Nef ELISA (Supplemental Fig. S1B).

### Nef ELISA

An in-house Nef ELISA was developed to quantify Nef associated to EVs. First, 96-well plates were coated with 0.25 μg of a mouse monoclonal anti-Nef antibody (Abcam, Waltham, MA; #ab42358) per well overnight. The coating antibody was diluted in coating buffer (Bio-Rad, Hercules, CA). After incubation overnight at room temperature, all wells were washed with ELISA washing buffer (Bio-Rad) and saturated with 300 μL of BSA blocking buffer (Bio-Rad) for 2 h at room temperature. Standards and samples were resuspended in 50 μL of Nef ELISA buffer (PBS 1X, 20 mM TRIS, 0.05% Tween 20, 0.2% BSA) supplemented with 0.5% Triton X-100. Samples were run in duplicate and quantified using the respective standard curve. The range of Nef (recombinant HIV-1_NL4-3_ Nef from Abcam, #ab63996) concentrations for standard curve was 1000 to 0.98 ng/mL. Following three washes with 300 μL of washing buffer, 50 μL of primary antibody (rabbit polyclonal anti-Nef antibody (Abcam, #ab63918) diluted in blocking buffer) was added to each well for 1 h at room temperature with gentle agitation. Wells were washed and 50 μL of secondary antibody [anti-rabbit horse peroxidase-conjugated antibody (SBI Biosciences, Palo Alto, CA)] was added to each well and incubated for 1 h at room temperature under gentle agitation. Wells were washed and 50 μL of TMB substrate (ThermoFisher Scientific, Waltham, MA) was added to each well. After incubation in the dark for 30 min under gentle agitation, the reaction was stopped by adding 50 μL of 2 N sulfuric acid in each well. OD was read immediately at 450 nm and 540 nm using a 96-well Synergy microplate reader (BioTek, Winooski, VT). For each sample, Nef concentrations were determined using 4P regression analysis of OD values in Prism.

### In Vivo injections

Sixteen- to twenty-week-old C57BL/6J male and female mice were anesthetized using isoflurane, and stereotaxic intracranial injection was performed. Five microliters of Nef or Ctrl EVs in sterile saline containing 10% mouse serum was infused at a rate of 7 nL/s into the right hemisphere corpus callosum 1.1 mm anterior and 1 mm lateral to bregma at a depth of 1.8 mm. At three days post injection, the mice were deeply anesthetized with avertin and then perfused with fixative, after which the tissue was processed for immunohistochemistry (IHC) or electron microscopy as described previously [55].

For immunohistochemical analysis, animals were perfused with 4% paraformaldehyde (PFA; EMS, 15710); brains were dissected, postfixed at 4 °C, equilibrated in 30% sucrose, embedded in Tissue-Tek® O.C.T. Compound (Sakura), and frozen at − 80 °C. Cryosections (11 µm) were prepared on a Leica CM1950 cryostat microtome.

For ultrastructural analysis via electron microscopy, mice were perfused with 4% PFA and 2% glutaraldehyde (EMS, 16010) in 0.1 M sodium cacodylate (EMS, 11653**)** adjusted to pH 7.4; 200 μm coronal sections were obtained with a vibratome (Campden Instruments, 5100 mz), postfixed in 1% OsO_4_ (EMS, 19192), dehydrated through a series of graded ethanol, incubated in saturated uranyl acetate, and infiltrated/embedded in a poly/Bed812 resin (EMS, 14900). One micrometer thick coronal sections were stained with toluidine blue, and areas for ultrathin EM sectioning were selected. Ultrathin coronal sections (120 nm) were placed on silicon wafers and carbon-taped in aluminum stubs for subsequent SEM imaging.

### Cerebellar slice culture

Primary cerebellar cultures were prepared from C57BL/6J mice at postnatal day 10 as previously described [55, 56]. Briefly, cerebella were embedded in 1% agarose and sagittally sectioned in ice-cold DPBS (Sigma‒Aldrich, G8769) supplemented with 5% glucose (Sigma‒Aldrich, G8769) at 300 μm using a Leica VT1000S vibrating microtome. Slices were immediately placed in 24-well plates on individual 0.4 μm 12 mm diameter Millicell-CM cell culture inserts (Millipore, PICM01250) and grown in 350 μL of cell culture medium containing 50% BME (Thermo Fisher, 21010046), 15% heat-inactivated horse serum (Thermo Fisher, 16050114), 25% Hank's solution (Sigma‒Aldrich, H4641), 0.5% glucose, 1% GlutaMAX™ Supplement (Thermo Fisher, 35050061), 1% penicillin‒streptomycin (Corning, 30-002-CI), N2, and 10 ng/mL PDGF-AA (Sigma‒Aldrich, P3076). The slices were incubated at 37 °C in 5% CO_2_. Half-volume media changes were made two days after plating. After 4 days, mouse cerebellar slices were treated with 10 µL of Nef or Ctrl EVs once daily for 2 or 4 days. The slices were fixed with 4% PFA for 15 min, delipidated in ice-cold 2.5% acid methanol for 15 min at − 20 °C, and subsequently processed for immunohistochemistry as described below.

### Mixed cell cultures

Primary mouse brain cells were obtained from the brains of C57BL/6J mice at postnatal day 3 using the Miltenyi Biotec Neural Tissue Dissociation Kit (Miltenyi Biotec, 130-093-231) according to the manufacturer’s instructions. Cells were plated on 12 mm coverslips coated with poly-L-lysine (PLL) (Sigma Aldrich, P1274) at 70,000 cells per well for immunostaining and on 6-well plates at 3 × 10^5^ cells per well for western blot and cytokine expression analysis. The culture media consisted of DMEM/F-12 supplemented with 1% FBS, 1% penicillin‒streptomycin, N2, and 10 ng/mL PDGF-AA. Cultures were grown for 5 days before treatment with 10 µL of Nef or Ctrl EVs for 48 h. For drug treatment, cells were cultured for 7 days prior to receiving once-daily treatment with 1 µM of AMS-55 (generously provided by Dr. Amol Kulkarni) or TO-901317 (Sigma‒Aldrich, 575310) for 3 consecutive days. Subsequently, the cells were treated with EVs for 48 h. Post-treatment, the media were collected for cytokine analysis, while cells underwent analysis via western blotting or immunohistochemistry, as described below.

### A2B5 + enriched cell cultures

Enriched populations of A2B5 + cells were isolated from the brains of C57BL/6J mice at postnatal day 5 via magnetic activated cell sorting with anti-A2B5 microbeads (Miltenyi Biotec, 130-093-392) according to the manufacturer’s instructions. The cells were plated on PLL-coated coverslips and differentiated into O4 + oligodendrocytes or GFAP + astrocytes. To promote OPC proliferation, A2B5 + cells were initially seeded at 10,000 cells per well and cultured for 24 h in medium supplemented with DMEM/F12 and 2X B-27 supplement (Thermo Fisher, 12587010), 2 mM l-glutamine (Gibco, 25030081), 20 ng/mL PDGF-AA and 20 ng/mL bFGF (PeproTech, 100-18B). The cells were then switched to oligodendrocyte differentiation medium containing DMEM/F12 supplemented with 2X B-27 supplement, 2 mM l-glutamine, 10 ng/mL PDGF, 30 ng/mL T3 (Sigma‒Aldrich, T63-97), and 10 ng/mL NT-3 (PeproTech, 450-03). Cultures were differentiated for 2 or 6 days before treatment with 10 µL of Nef or Ctrl EVs for 48 h and then analyzed via immunohistochemistry. To promote astrocyte differentiation and proliferation, A2B5 + cells were initially seeded at 10,000 cells per well and cultured in “astrocyte media”, DMEM/F-12 supplemented with 5% FBS, 1% penicillin‒streptomycin, N2, and 10 ng/mL PDGF-AA. After 3 days, the cells were treated with 10 µL of Nef or Ctrl EVs for 48 h and then analyzed via immunohistochemistry.

### Immunohistochemistry

Brain cryosections were processed for immunohistochemistry as described previously [55], frozen sections were dried at room temperature for 1 h and rehydrated in 1 × PBS for 5 min. Samples subjected to MBP analysis were treated for 20 min at − 20 °C with ice-cold 2.5% acid methanol. All the samples were then incubated for 1 h at room temperature in 10% normal goat serum (NGS) and 0.3% Triton™ X-100 (Sigma‒Aldrich) in 1 × PBS and incubated overnight at 4 °C with primary antibodies (Supplementary Table 1), followed by incubation with Alexa Fluor-conjugated secondary antibodies (1:500) for 1 h at room temperature (both primary and secondary antibodies were diluted in 10% NGS and 0.3% Triton in 1 × PBS). Sections were counterstained with DAPI (1:3000; Thermo Fisher, 46190) and mounted in ProLong™ Gold Antifade Mountant (Invitrogen, P36934).

Processing samples from slice cultures was performed as described previously [57]. After the slices were fixed with 4% PFA and delipidated in 2.5% acid methanol, the slices were blocked first with a primary blocking solution (0.1% Triton, 15% NGS, 10% bovine serum albumin (BSA; Fisher, BP9706100), diluted in PBS) for 1.5 h at room temperature and then with a secondary blocking solution (0.1% saponin, 5% NGS, 5% BSA, diluted in PBS) for 3 h at room temperature. The slices were incubated at 4 °C overnight with primary antibodies, incubated with the appropriate Alexa Fluor-conjugated secondary antibodies (1:500) and DAPI (1:5,000) for 3 h at room temperature (primary and secondary antibodies diluted in 0.1% saponin and 5% NGS with 1xPBS), and then mounted in VECTASHIELD® PLUS Antifade Mounting Medium (Vector Laboratories, H-1900).

Cell cultures were stained either live or after fixation. For live staining with anti-O4 IgM, coverslips were incubated at 37 °C with primary antibody for 30 min, followed by incubation with secondary antibody for 15 min (both primary and secondary antibodies were diluted in 10% NGS with warm DMEM). The cells were fixed with 2.5% acid methanol for 20 min at − 20 °C and then labeled for immunohistochemistry. Coverslips were then incubated for 1 h at room temperature with primary antibodies (Table 1) followed by incubation with Alexa Fluor-conjugated secondary antibodies (1:500) for 1 h at room temperature (both primary and secondary antibodies were diluted in 10% NGS and 0.3% Triton in 1xPBS). Sections were counterstained with DAPI (1:3000) and mounted in VECTASHIELD® PLUS Antifade Mounting Medium.

**Table 1:** Antibodies used for immunohistochemistry

Antibodies	Vendor	Catalog #	Clone	Dilution	Use
Mouse anti-myelin basic protein (MBP)	Biolegend	836504	SMI-99	1:500	Detection of myelin
Mouse anti-APC	Millipore	MABC200	CC1	1:500	Identify mature oligodendrocytes
Rat anti-GFAP	Invitrogen	13-0300	2.2B10	1:500	Detection of astrocytes
Rabbit anti-Vimentin	Abcam	ab92547	EPR3776	1:500	Detection of astrocytes, glial precursors, and ependymal cells
Rabbit anti-Iba1	Wako Chemicals	019-19741	polyclonal	1:500	Detection of microglia/macrophages
Chicken anti-Neurofilament M	Biolegend	822701	Poly28227	1:500	Detection of axons
mouse anti-O4 IgM	R&D Systems	MAB1326	04	1:50	Identify oligodendrocytes
Goat anti-mouse Alexa 594	Invitrogen	A11032	–	1:500	Secondary
Goat anti-mouse Alexa 488	Invitrogen	A11029	–	1:500	Secondary
Goat anti-rabbit Alexa 594	Invitrogen	A11012	–	1:500	Secondary
Goat anti-rabbit Alexa 488	Invitrogen	A11008	–	1:500	Secondary
Goat anti-rat Alexa 594	Invitrogen	A11007	–	1:500	Secondary
Goat anti-rat Alexa 488	Invitrogen	A11006	–	1:500	Secondary
Goat anti-chicken Alexa 647	Invitrogen	A21449	–	1:500	Secondary
Goat anti-mouse IgM Alexa 488	Invitrogen	A21042	–	1:300	Secondary

### TUNEL assay

Apoptotic cells in mixed cultures were detected with the Click-iT™ Plus TUNEL Assay Kit for In Situ Apoptosis Detection (Invitrogen, C10617) according to the manufacturer’s protocol. Briefly, cells were grown on coverslips, fixed with 2.5% acid methanol for 20 min at − 20 °C and permeabilized with 0.25% Triton X-100 for 20 min at room temperature. Coverslips were then incubated at 37 °C with TdT reaction buffer for 10 min, followed by incubation with the TdT reaction mixture for 1 h. After rinsing with 3% BSA in PBS, the coverslips were incubated with Click-iT™ Plus TUNEL reaction cocktail for 30 min at 37 °C. After washing with 3% BSA in PBS, the coverslips were counterstained with DAPI (1:3000) and mounted in VECTASHIELD® PLUS Antifade Mounting Medium.

### Electron microscopy and analysis

To determine the extent of the lesions and relevant alterations in myelin ultrastructure, axonal integrity, and perivascular astrocytes in the corpus callosum after Nef (n = 2) or Ctrl (n = 3) EV injections, scanning electron microscopy (SEM) analysis was performed at The George Washington University (GW) Nanofabrication and Imaging Center (GWNIC) using an FEI Helios NanoLab 660 DualBeam SEM (Thermo Fisher). To maximize the backscatter electron collection, we used a concentric backscattering detector (CBS) in immersion mode to acquire high-resolution SEM images. The acquisition conditions included 2 kV with a beam landing current ranging from 0.1 to 0.4 nA and a working distance of 4 mm. For imaging, we used a low-magnification tile area, including the cortex, corpus callosum, and hippocampus (600 ×), as a navigation map to identify the lesion location (MAPS software). Then, high-resolution (12,000 ×) imaging was performed in the focus area at a 5 ms dwell time, 34.5 mm horizontal field of view, and a pixel width of 11.2413 nm.

### Fluorescence microscopy and analysis

Fluorescence and phase contrast microscopy were performed by an individual blinded to the experimental conditions using a Leica DM 5500 microscope with a Hamamatsu ORCA-R2 camera, and the results were analyzed with ImageJ (NIH). In vivo 20 × tile scan images of the ipsilateral and contralateral corpus callosum were obtained from 3 to 5 mice per condition. Regions of interest (ROIs) of the ipsilateral hemisphere were identified as 400-to-500-micron wide sections of the corpus callosum centered at the injection site; corresponding ROIs were then made in the contralateral hemisphere. For GFAP and vimentin expression analysis, single 40 × images of the corpus callosum were obtained directly at the injection site, and Mander’s correlation and Pearson’s overlap coefficients were estimated employing the ImageJ Fiji JaCoP plugin.

Ex vivo slice culture images were captured at 20 × from 2 to 3 sections per treatment condition. All the images were processed with the Leica Blind Deblur tool. The NF + /MBP- axon length was measured with the ImageJ line tool. The MBP-stained area was calculated as a percentage of the total tissue area.

In vitro images were captured at 20 × or 40 ×, and the cells were counted in ImageJ using a cell counter tool. For experiments involving O4 + oligodendrocytes, only live cells with intact nuclei and cell bodies were counted. “Normal branching” was defined as the presence of complex branching processes without disrupted or severely diminished immunostaining. Cell damage in purified oligodendrocyte cultures was indicated by the presence of DAPI + chromatin fragments, which are hallmarks of apoptosis [58], overlaying injured cells (when analyzed by phase contrast microscopy) and disrupted oligodendrocytes (by O4 + immunofluorescence) (Supplemental Fig. 2). DAPI + fragments were identified in ImageJ using watershed separation and particle tools with an area up to 5 microns. For IBA1 + microglia, highly ramified cells were defined as those possessing one or more projections with a length greater than 1.5 × that of the cell body. GFAP + astrocytes were categorized based on morphology as follows: (i) fibrous with an amorphous shape, (ii) bipolar with a single defined projection from the cell body, or (iii) multipolar with two or more projections from the cell body. Images for analysis by TUNEL were captured at 10 ×, and randomized ROIs with an area of 0.04 mm^2^ were generated to count TUNEL + cells. All in vitro experiments were run on coverslips in duplicate or in triplicate, with 2–3 images per replicate.

### Cytokine measurement

The cell culture supernatant was collected, and cytokine protein expression measured with a mouse atherosclerosis antibody array (Abcam, #ab169807) according to the manufacturer’s instructions. The membrane array can simultaneously detect 22 factors implicated in atherosclerosis, including those commonly upregulated in individuals with HAND. First, the total protein concentrations of the culture supernatants were determined by a BCA assay (Thermo Fisher, #23225) to confirm equivalent protein levels between treatment conditions. The membrane arrays were incubated with 1X blocking buffer for 30 min at room temperature and then with 1 mL of supernatant from the EV-treated cultures overnight. The membranes were incubated overnight with biotin-conjugated anti-cytokine antibodies followed by incubation overnight with HRP-conjugated streptavidin. All the incubations were performed at 4 °C with gentle shaking on a platform shaker. The membranes were imaged with a GeneGnome XRQ chemiluminescence imaging system (Syngene). Densitometry data were extracted from array images using ImageJ software (NIH). After background subtraction, each array was normalized to its positive control signal, and the normalized signal intensities were compared between treatment conditions to determine relative differences in cytokine expression.

### Western blot

Automated capillary western immunoblots were performed using the ProteinSimple Jess™ Simple Western instrument (Bio-Techne) according to the manufacturer’s instructions as described previously [59]. Cells were homogenized in NET buffer (1% NP-40 lysis buffer: 150 mM NaCl, 1% NP40, 50 mM Tris HCl at pH 8.0) containing protease and phosphatase inhibitors (Thermo Fisher). Cell lysates were centrifuged at 700*g* for 5 min. Four microliters of cleared cell lysate (1 μg/μL protein) were mixed with 1 μL of 5 × master mix containing fluorescent molecular weight markers and 200 mM dithiothreitol. For ABCA1 expression analysis, the mix was incubated at 37 °C for 15 min. The 66–440-kDa Jess separation module (Bio-Techne, #SM-W008) was used for electrophoresis. To prepare the assay plate for ABCA1 analysis, we loaded a molecular weight ladder, protein samples, KPL blocking buffer (SeraCare Life Sciences, #5920-0004), total protein quantification reagent, rabbit polyclonal anti-ABCA1 (1:40; Abcam, #ab63918), rabbit polyclonal anti-GAPDH (1:100; Sigma, #G9545), secondary HRP conjugated anti-rabbit antibody (Bio-Techne, #042-206) and chemiluminescent substrate according to the plate diagram. After plate loading, the separation, electrophoresis, total protein quantification, and immunodetection steps were conducted in the fully automated Jess system. Digital image of chemiluminescence of the capillary was captured with Compass Simple Western software (version 5.1.0, Protein Simple) that automatically calculated heights (chemiluminescence intensity), area, and signal/noise ratio. Results were visualized as electropherograms representing the peak of chemiluminescence intensity and as lane view from the signal of chemiluminescence detected in the capillary.

### Data analysis

All the values are presented as the average ± SEM. Student’s t test, one-way ANOVA, or two-way ANOVA was performed where appropriate using GraphPad Prism software. p < 0.05 was considered to indicate statistical significance.

## Results

### Nef EVs disrupt CNS myelin in vivo

**Fig. 1:**
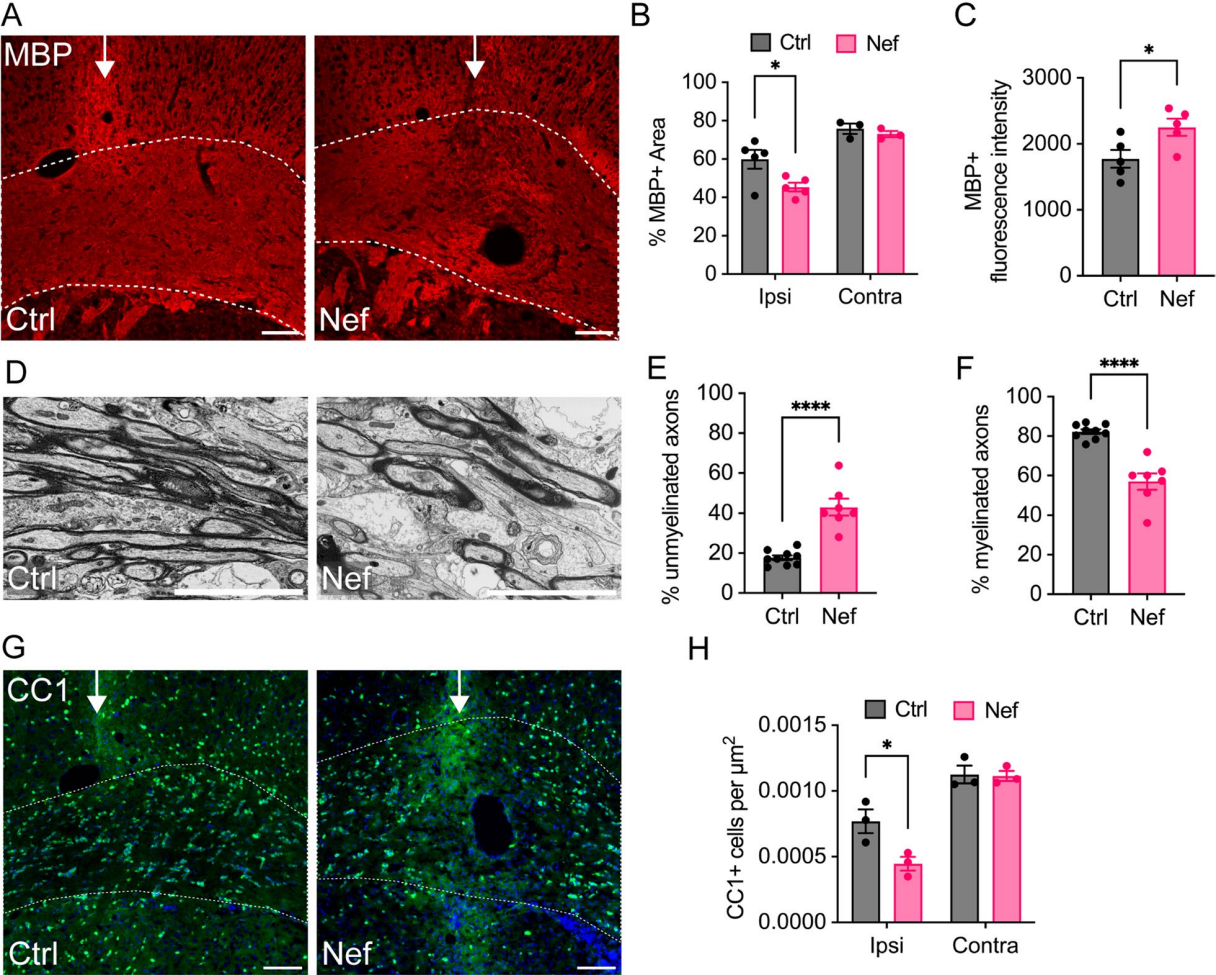
Effects of Nef EVs on myelination of axons in vivo. Mice were intracranially injected with either Ctrl or Nef EVs. At 3 days post-injection, brain tissue was processed for analysis using immunohistochemistry (IHC) or scanning electron microscopy (SEM). Images depict representative fields from the corpus collosum at the injection site. **A** Representative fluorescence images of myelin (MBP, red). **B** Quantification of the percentage of MBP + immunofluorescence area within the ipsilateral (ipsi) and contralateral (contra) corpus callosum. **C** Quantification of MBP + fluorescence intensity within the corpus callosum at the injection site. **D** Representative SEM images within the corpus callosum lesion. **E** Quantification of the percentage of unmyelinated axons within the corpus callosum lesion by SEM. **F** Quantification of the percentage of myelinated axons within the corpus callosum lesion by SEM. **G** Representative fluorescence images of mature oligodendrocytes (CC1, green) and nuclei (DAPI, blue). **H** Quantification of CC1 + cells per µm^2^ within the ipsilateral and contralateral corpus callosum. The white arrow indicates the injection site and the dotted line indicates the border of the corpus callosum. For IHC, n = 3–5 mice per group, scale bar is 100 µm. For SEM, n = 7–9 regions of interest (ROIs) from 2 to 3 animals per group, scale bar is 5 µm. *p < 0.05; **p < 0.01; ***p < 0.001; ****p < 0.0001

To determine whether Nef EVs can contribute to the oligodendrocyte and white matter deficits observed in patients with HAND, Nef-containing and control EVs (Nef EVs and Ctrl EVs, respectively), purified from empty vector-transfected (Ctrl EVs) or from Nef-transfected (Nef EVs) HEK293T cells as previously described [60], were injected into the white matter of the mouse brain (corpus callosum of the right (ipsilateral) hemisphere) via stereotactic surgery. Tissue was collected 3 days post injection for analysis by immunohistochemistry and electron microscopy. Disruption of myelin in EV-injected white matter was assessed by the expression of myelin basic protein (MBP) within the corpus callosum at the injection site (Fig. 1A). Compared to that in the Ctrl group, the total MBP + immunofluorescence within the Nef EV-injected white matter was significantly lower in the ipsilateral corpus callosum (Fig. 1B), consistent with a localized myelin lesion. This change was accompanied by a focal increase in the mean MBP + fluorescence intensity (Fig. 1C), a common finding within myelin lesions due to increased antibody binding in the demyelinating tissue [61]. Electron microscopy (EM) examination of the myelin ultrastructure at the injection site also revealed an increase in the number of unmyelinated axons and perturbation of myelin within Nef EV-injected white matter, which was not observed in Ctrl EV-injected tissue (Fig. 1D). Compared to those in the control group, the white matter in the Nef EV-injected group displayed a statistically significant 2.4-fold increase in the number of unmyelinated axons, which had a relatively normal ultrastructural appearance (Fig. 1E). Conversely, there was a significant 31% decrease in myelinated axons (Fig. 1F). Together, these findings suggest that Nef EVs directly disrupt CNS myelin sheaths in the absence of overt axonal degeneration. This result resembles that reported for the Plp1-transgenic mouse model of Pelizaeus-Merzbacher disease [60], and suggests that demyelination, at least in the short term, does not lead to neuronal death.

Injection of Nef EVs was also associated with a significant local reduction (by 42%) in the density of CC1 + (a marker of mature oligodendrocytes) cells within the ipsilateral corpus callosum after 3 days (Fig. 1G, H), indicating that Nef EVs may cause the loss of mature oligodendrocytes in vivo. No change in the density of myelin or oligodendrocytes was detected in regions of the corpus callosum contralateral to the site of EV injection (Fig. 1B, H), indicating that the effects of Nef EVs on oligodendrocytes and myelin are localized to sites of direct Nef EV exposure.

### Nef EVs disrupt astrocytes and microglia in vivo

**Fig. 2:**
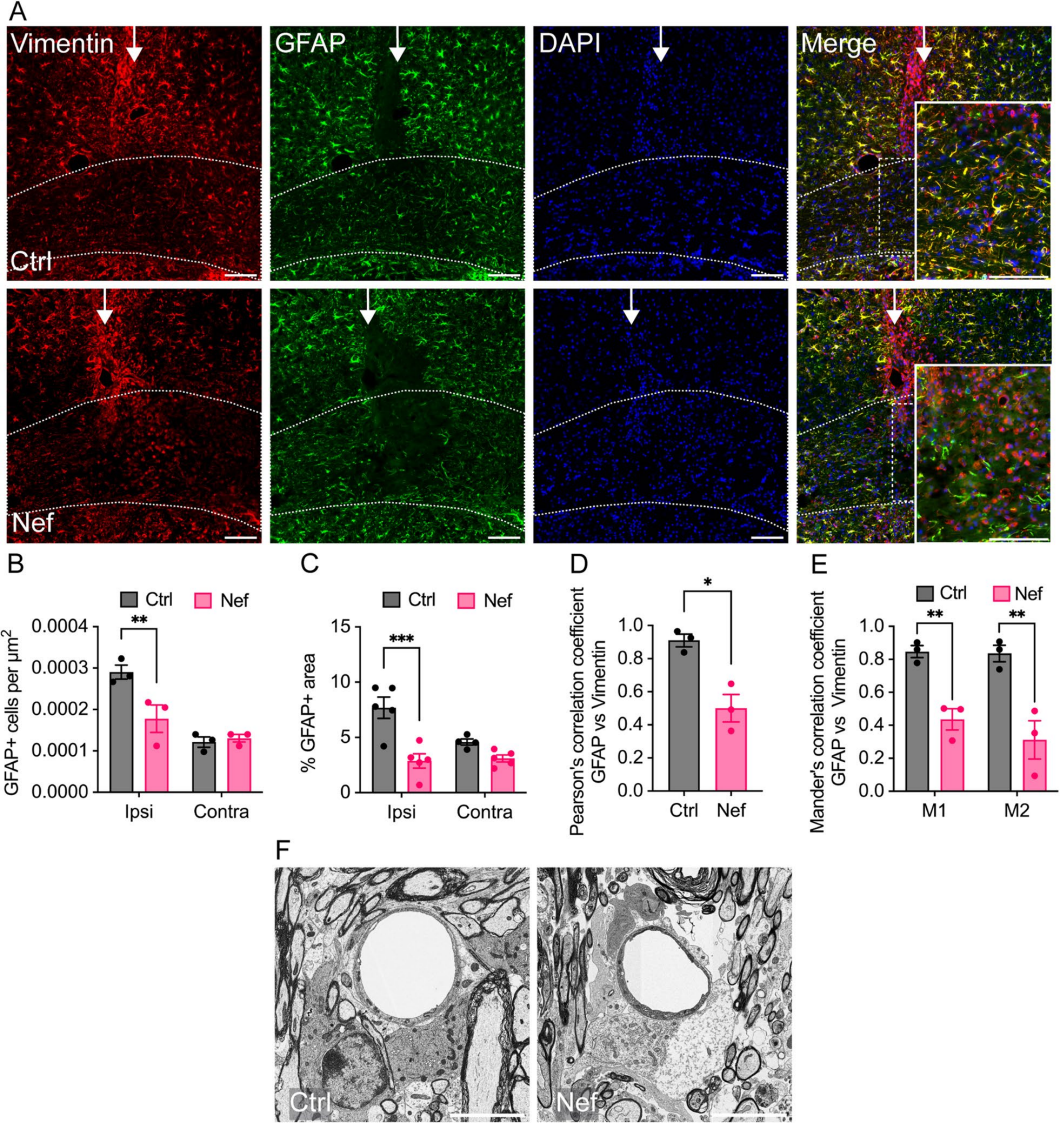
Effects of Nef EVs on astrocytes in vivo. Mice were intracranially injected with either Ctrl or Nef EVs. At 3 days post injection, brain tissue was processed for analysis using immunohistochemistry (IHC) or scanning electron microscopy (SEM). The images display representative fields from the corpus collosum at the injection site. **A** Representative fluorescence images of vimentin (red), GFAP (green), and nuclei (DAPI). Higher magnification insets illustrate the loss of GFAP within the lesion area. **B** Quantification of GFAP + cells per µm^2^ within the ipsilateral (ipsi) and contralateral (contra) corpus callosum at the lesion. **C** Quantification of the percentage of GFAP + immunofluorescence area within the ipsilateral and contralateral corpus callosum at the lesion. **D**, **E** Analysis of colocalization of GFAP and vimentin within the ipsilateral corpus callosum at the lesion. *M1*: fraction of GFAP in the vimentin area, and *M2*: fraction of vimentin in the GFAP area. **F** Representative SEM images of perivascular astrocytes within the corpus callosum lesion. The white arrow indicates the injection site and the dotted line indicates the border of the corpus callosum. For IHC, n = 3–5 mice per group, scale bar is 100 µm. For SEM, scale bar is 5 µm. *p < 0.05; **p < 0.01; ***p < 0.001; ****p < 0.0001

We have previously shown that astrocytes are required for maintaining myelin integrity in the adult CNS [55], and other studies have reported that Nef expression leads to downregulation of the gene expression of the major astrocytic protein glial fibrillary acidic protein (GFAP) [62]. Since disruptions to astrocytes may contribute to Nef EV-mediated demyelination, we examined astrocytes in the brains of EV-injected mice via immunohistochemistry and EM. Injection of Nef EVs into the right hemisphere corpus callosum resulted in a marked localized loss of GFAP + cells at the injection site (Fig. 2A), with a 30% decrease in the number of GFAP + cells (Fig. 2B) and a 63% decrease in GFAP + immunofluorescence (Fig. 2C) within the myelin lesion area compared with those in Ctrl EV-injected animals. The loss of GFAP + cells was correlated with perturbations in the ultrastructural morphology of astrocytes in the lesion area, including swelling of perivascular astrocyte processes (Fig. 2F). There was no significant difference in GFAP + cell density or staining in the contralateral corpus callosum, indicating that this effect on GFAP + expression in astrocytes was localized to the area of the injection.

To further characterize the effect of Nef-derived EVs on astrocytes, we analyzed the relationship between GFAP (a mature astrocyte marker) and vimentin (a panglial and immature astrocyte marker implicated in reactive astrogliosis [63, 64]) in the lesion area of the ipsilateral corpus callosum via colocalization analysis. In all analyses, Pearson’s correlation and Mander’s overlap coefficients were estimated employing the ImageJ Fiji JaCoP plugin. All colocalization coefficients were significantly greater for the Ctrl EV-injected white matter than for the Nef EV-injected white matter (Fig. 2D, E). The mean Pearson’s correlation coefficient was approximately halved for Nef EV- relative to that for Ctrl EV-injected white matter (0.91 to 0.50, a 45% decrease), denoting a shift from a strong to a moderately positive correlation between GFAP and vimentin signals. Furthermore, Mander’s *M1* and *M2* coefficients ranged between 0.7 and 1.0 in the Ctrl EV-injected white matter, indicating the strong overlap of GFAP fractions in the vimentin area (*M1*) and vimentin fractions in the GFAP area (*M2*); Mander’s *M1* and *M2* coefficients in the Nef EV-injected white matter ranged between 0 and 0.5, indicating only a weak to moderate overlap of GFAP fractions in the vimentin area (*M1*) and vimentin fractions in the GFAP area (*M2*). The overall correlation and co-occurrence coefficient results suggested that GFAP and vimentin overlapped less in Nef-EV-injected white matter than in control white matter, indicating that Nef EVs induced a change in the expression of astrocytic markers implicated in cell differentiation and activation in the setting of a lesion.

**Fig. 3:**
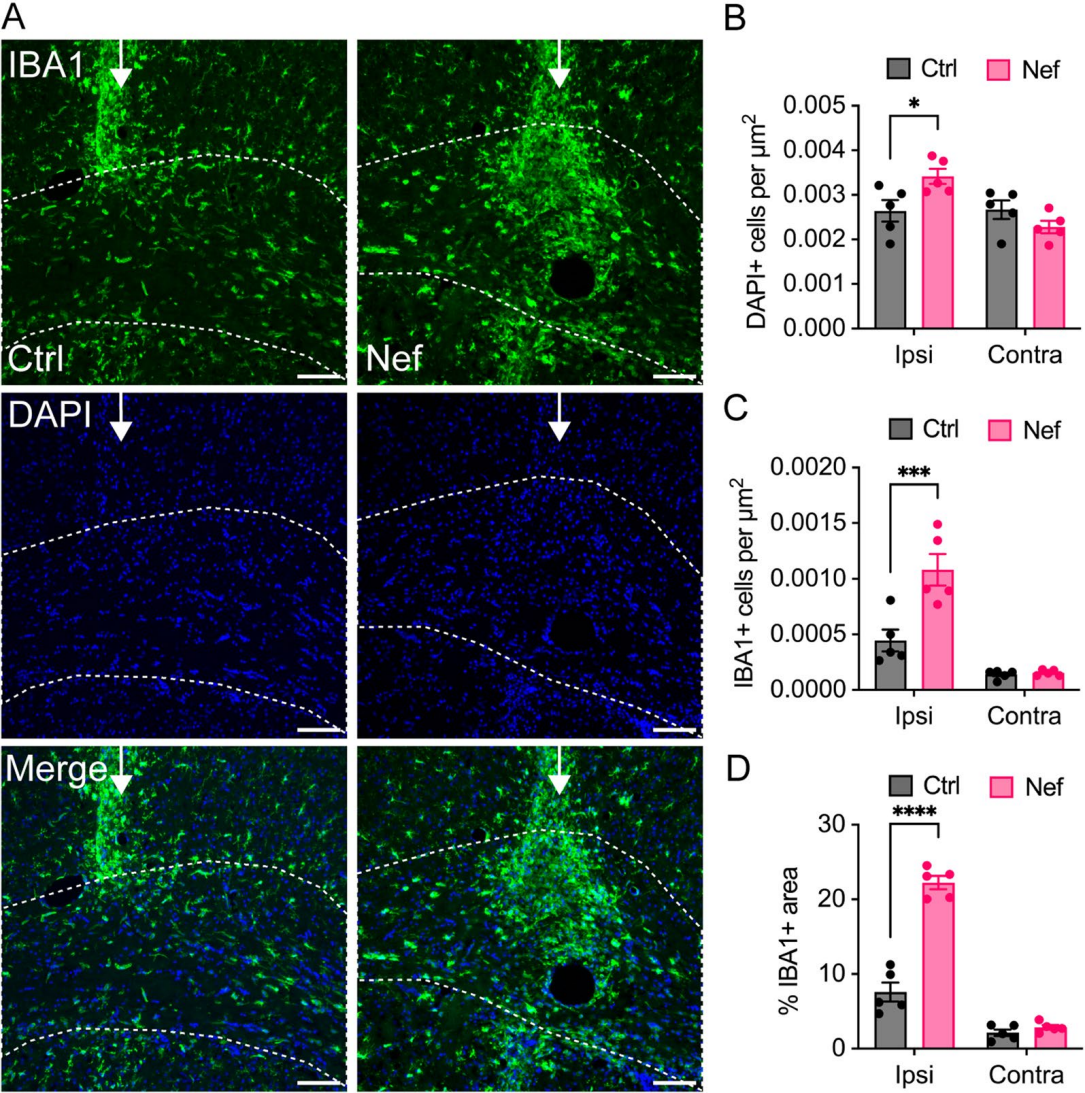
Effects of Nef EVs on microglia in vivo. Mice were intracranially injected with Ctrl or Nef EVs. At 3 days post injection, brain tissue was processed for analysis by immunohistochemistry. The images depict representative fields from the corpus collosum at the lesion site. **A** Representative fluorescence images of microglia/myeloid cells (IBA1, green) and nuclei (DAPI, blue). **B** Quantification of DAPI + immunofluorescence per µm^2^ within the ipsilateral (ipsi) and contralateral (contra) corpus callosum. **C** Quantification of IBA1 + cells per µm^2^ within^2^ the ipsilateral and contralateral corpus callosum. **D** Quantification of the percentage of IBA1 + immunofluorescence within the ipsilateral and contralateral corpus callosum at the injection site. N = 5 mice per group. The white arrow indicates the injection site and the dotted line indicates the border of the corpus callosum. For IHC, n = 5 mice per group, scale bar is 100 µm. *p < 0.05; **p < 0.01; ***p < 0.001

Next, we examined brain tissue from EV-injected mice for evidence of neuroinflammation, which generally accompanies HIV-mediated brain disease [65] and can promote or reflect active demyelination. Previous studies showed that peripheral injection of Nef EVs in vivo was associated with elevated plasma levels of IL-6 and TNFα [60], suggesting that Nef EVs can promote inflammation. To assess the inflammatory response to direct CNS injection, the ipsilateral and contralateral corpus callosum at the injection site were analyzed for microglial infiltration and activation (Fig. 3). Compared to Ctrl EV-injected tissue, ipsilateral white matter from Nef EV-injected brains showed a 29% increase in DAPI positive cells (Fig. 3A, B) accompanied by a 2.9-fold increase in microglia/myeloid cell activation (extent of IBA1 + staining area) and a 2.4-fold increased density of activated cells (Fig. 3C, D). Taken together, these data revealed increased infiltration and activation of IBA1 + microglia within white matter injected with Nef EVs, indicating that an increased inflammatory response may contribute to Nef-mediated demyelination. There was no difference in the number of DAPI + cells, IBA1 + cell density, or IBA1 + activation in the contralateral corpus callosum, indicating that this inflammatory response was localized to the site of injection.

### Nef EVs disrupt myelin in cerebellar slice cultures ex vivo

**Fig. 4:**
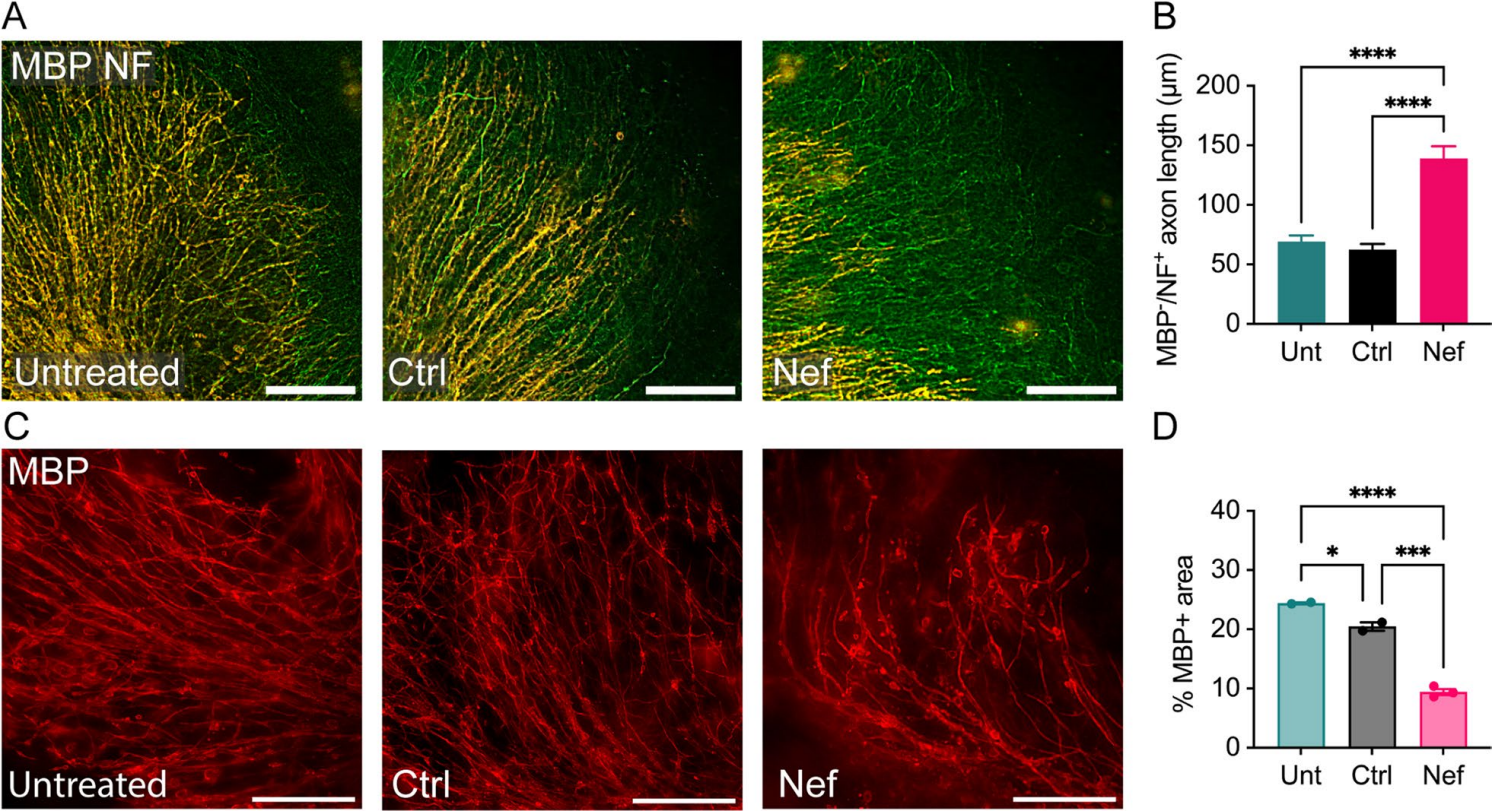
Effects of Nef EVs on myelin in cerebellar slice cultures. Cerebellar slice cultures derived from C57BL/6J mice at postnatal day 10 were cultured for 4 days and subsequently treated with either Ctrl or Nef EVs once daily for 2 or 4 days. **A** Representative fluorescence images depict myelin (MBP, red) and axons (NF, green) 2 days after treatment. **B** Quantification of unmyelinated axon length 2 days post-treatment, measured as MBP-/NF + axon length in µm. Axons were measured on a single slice each for untreated (n = 33 axons), Ctrl EV-treated (n = 23 axons), and Nef EV-treated (n = 31 axons) groups. **C** Representative fluorescence images display myelin (MBP, red) 4 days after treatment. **D** Quantification of the percentage of MBP + immunofluorescence per field. N = 2–3 sections per treatment condition. Scale bar is 100 µm. *p < 0.05; **p < 0.01; ***p < 0.001; ****p < 0.0001

To assess the effects of Nef EVs on CNS myelin integrity in the absence of immune cell infiltration, cerebellar slice cultures from C57BL/6J mice at postnatal day 10 were grown on tissue culture inserts for 4 days and treated once daily with Nef EVs or Ctrl EVs for 2 or 4 days. The concentration of EV-associated Nef added to the cultures was 241.4 pg/mL, approximately tenfold less than the concentration of Nef found in the blood of more than 50% of ART-treated HIV-infected individuals with undetectable viral load [43]. After 2 days, the length of the unmyelinated axons in the Nef EV-treated slices significantly increased (twofold relative to that in the untreated slices and 2.2-fold relative to that in the Ctrl EV-treated slices) (Fig. 4A, B). After 4 days, sections from Nef EV-treated slices had significantly less MBP expression than their counterparts (61% decrease relative to untreated, and 54%—relative to Ctrl EV-treated) with visibly disrupted myelin sheaths (Fig. 4C, D). These results suggest that less total myelin was present in cerebellar tissue exposed to Nef EVs. Taken together, these findings indicate that Nef EVs induce changes in myelin structure and integrity in the absence of an infiltrating inflammatory cell response and axonal degradation and that these effects increase over time with continued exposure to Nef EVs.

### Nef EVs disrupt oligodendrocytes in mixed primary mouse brain cultures

**Fig. 5:**
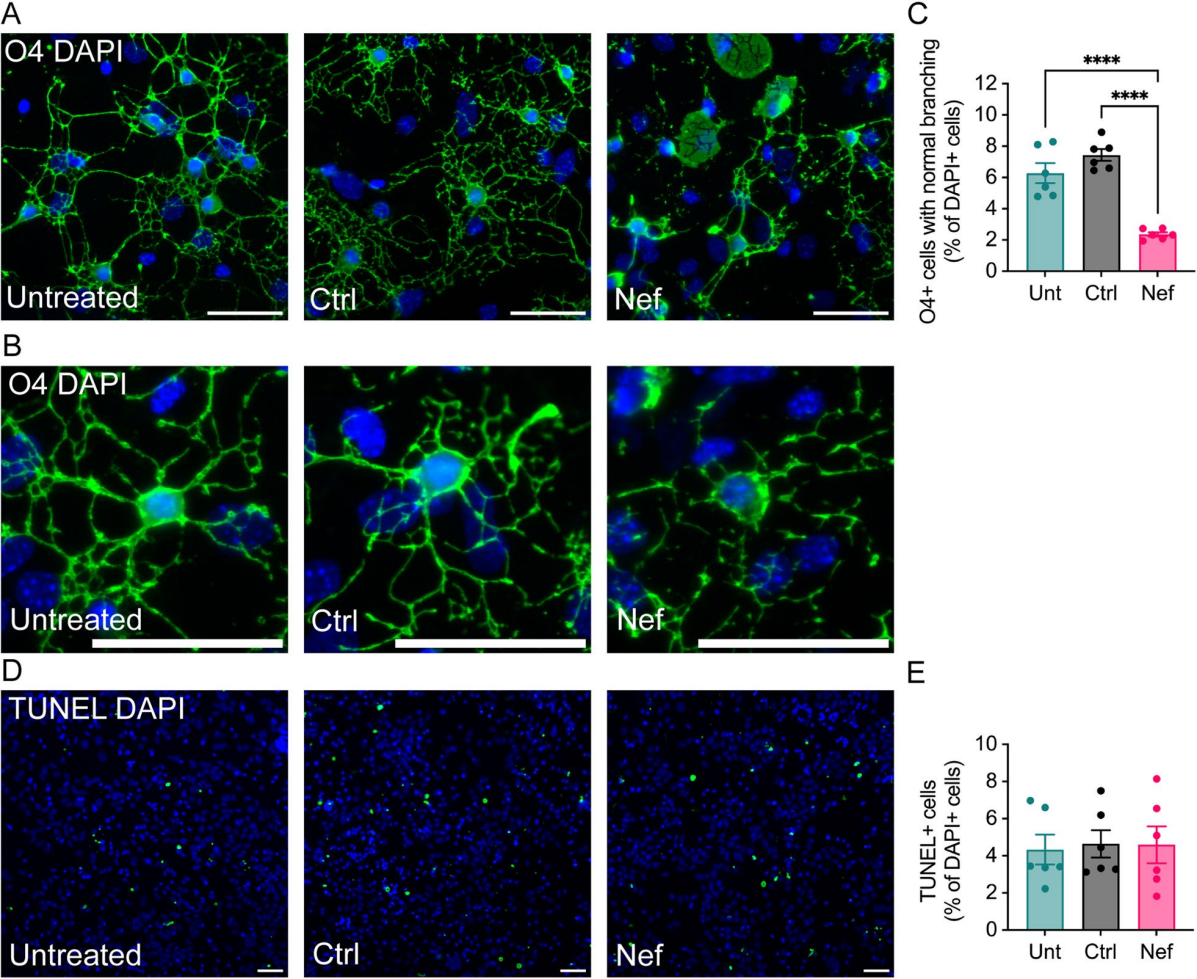
Effects of Nef EVs on oligodendrocytes in primary brain cultures. Mouse primary brain cells were collected at postnatal day 3 and grown for 5 days in media to promote oligodendrocyte growth. Subsequently, the cells were treated with either Ctrl or Nef EVs for 48 h. **A**, **B** Untreated (Unt) cultures served as negative controls. Representative fluorescence images illustrate oligodendrocytes (O4, green) and nuclei (DAPI, blue). Higher magnification images displaying branching oligodendrocyte morphology are presented in (**B**). **C** Quantification of O4 + oligodendrocytes with clear immunostaining and normal branching as a proportion of total DAPI + cells. **D** Representative fluorescence images of the TUNEL assay. **E** Quantification of the TUNEL assay**.** Scale bar is 50 µm. N = 6, ****p < 0.0001

Oligodendrocyte-specific expression of recombinant Nef affects proper differentiation of oligodendrocytes in vitro [28]. To determine the effects of extracellular Nef EVs on oligodendrocytes in vitro, mixed brain cultures were obtained from neonatal C57BL/6J mice at postnatal day 3, grown in media promoting oligodendrocyte development for 5 days in vitro, and then were treated once daily with Nef EVs or Ctrl EVs for 2 days. Oligodendrocytes were detected using a monoclonal antibody against O4 since O4 expression begins shortly after lineage commitment and is associated with the duration of oligodendrocyte differentiation [28, 66]. In the presence of Nef EVs, O4 + cells were visibly disrupted, and their morphology was altered (Fig. 5A, B); this disruption included a decreased number and disorder of the branching process. We quantified the number of oligodendrocytes with normal branching and found that Nef EV treatment resulted in a decrease in the number of branching O4 + oligodendrocytes (62% relative to that in the untreated group and 68% relative to that in the Ctrl EV-treated group) (Fig. 5C). The total number of O4 + cells was not significantly different in the presence of Nef EVs, and there was no significant change in TUNEL immunoreactivity between treatment conditions (Fig. 5D, E), suggesting that Nef EVs were not immediately toxic to oligodendrocytes but rather compromised their ability to generate or maintain normal cellular architecture in the presence of other neural cells.

### Nef-EVs disrupt oligodendrocytes in A2B5 + -enriched cultures

To assess the effects of Nef EV treatment on oligodendrocyte lineage cells, A2B5 + oligodendrocyte progenitor cells were isolated from C57BL/6J mice at postnatal day 5 and differentiated into O4 + oligodendrocytes for 2 or 6 days before treatment with Ctrl or Nef EVs once daily for 2 days (Supplemental Fig. S3A, E). After the cells were cultured for 2 days in oligodendrocyte differentiation medium prior to EV treatment, there was no significant change in the percentage of O4 + oligodendrocytes between the treatment groups (Supplemental Fig. S3B). However, Nef EV treatment resulted in a significant increase in the number of chromatin fragments with a diameter less than 5 µm. Notably, this effect was observed throughout the culture (Supplemental Fig. S3C) but was more pronounced within cell clusters (Supplemental Figs. S3A, C). Chromatin condensation and degradation of genomic DNA into fragments are common morphological markers of apoptosis [58]; accordingly, phase and fluorescence imaging of A2B5 + -enriched oligodendrocyte cultures revealed that DAPI + fragments overlayed injured cells (via phase contrast microscopy) and disrupted oligodendrocytes (via O4 + immunofluorescence) (Supplemental Fig. S2).

To determine the effect of Nef EVs on more mature oligodendrocytes, EVs were added to A2B5 + OPCs after 6 days of differentiation (Supplemental Fig. S3E). In addition to an increase in DAPI + fragments, we observed a significant decrease in the percentage of O4 + cells in Nef EV-treated cultures, with significant disruptions to oligodendrocyte morphology (Supplemental Fig. S3E-G). Taken together, these results suggest that in the absence of other neural cells, Nef EVs directly damage mature oligodendrocytes, and the lack of effect on the total cell number in younger cultures may reflect continued generation of O4 + cells from the original A2B5 progenitors.

### Nef EVs alter microglia and astrocytes in mixed primary mouse brain cultures

Since Nef-mediated demyelination and oligodendrocyte damage in vivo are associated with changes in astrocytes and microglia, we also examined the effects of Nef EVs on these cell types in primary mouse brain cultures. Treating mixed brain cultures for 48 h with Nef EVs resulted in a shift in the morphology of IBA1 + microglia/myeloid cells toward a highly ramified morphology (25% increase relative to that of the untreated group; 42% increase relative to that of the Ctrl EV-treated group; Supplemental Fig. S4A-B), with no change in the number of IBA1 + cells across all treatment conditions (Supplemental Fig. S4C). This shift in microglial morphology may indicate a change in phenotype toward an activated state. To further determine whether Nef EVs potentiate a proinflammatory response in CNS cells that could contribute to oligodendrocyte damage, we analyzed the supernatant of primary brain cultures treated with Ctrl or Nef EVs for the presence of 22 cytokines. A cytokine array did not reveal significant changes in protein expression between Ctrl and Nef EV-treated cultures (Supplemental Fig. S5), indicating that Nef EVs alone do not induce an inflammatory response in culture and that oligodendrocyte damage is not dependent on the expression of the cytokines assayed. These results are in line with our previous studies on monocyte-derived macrophages in vitro, which indicated that Nef alone is unable to induce a cytokine response [60].

**Fig. 6:**
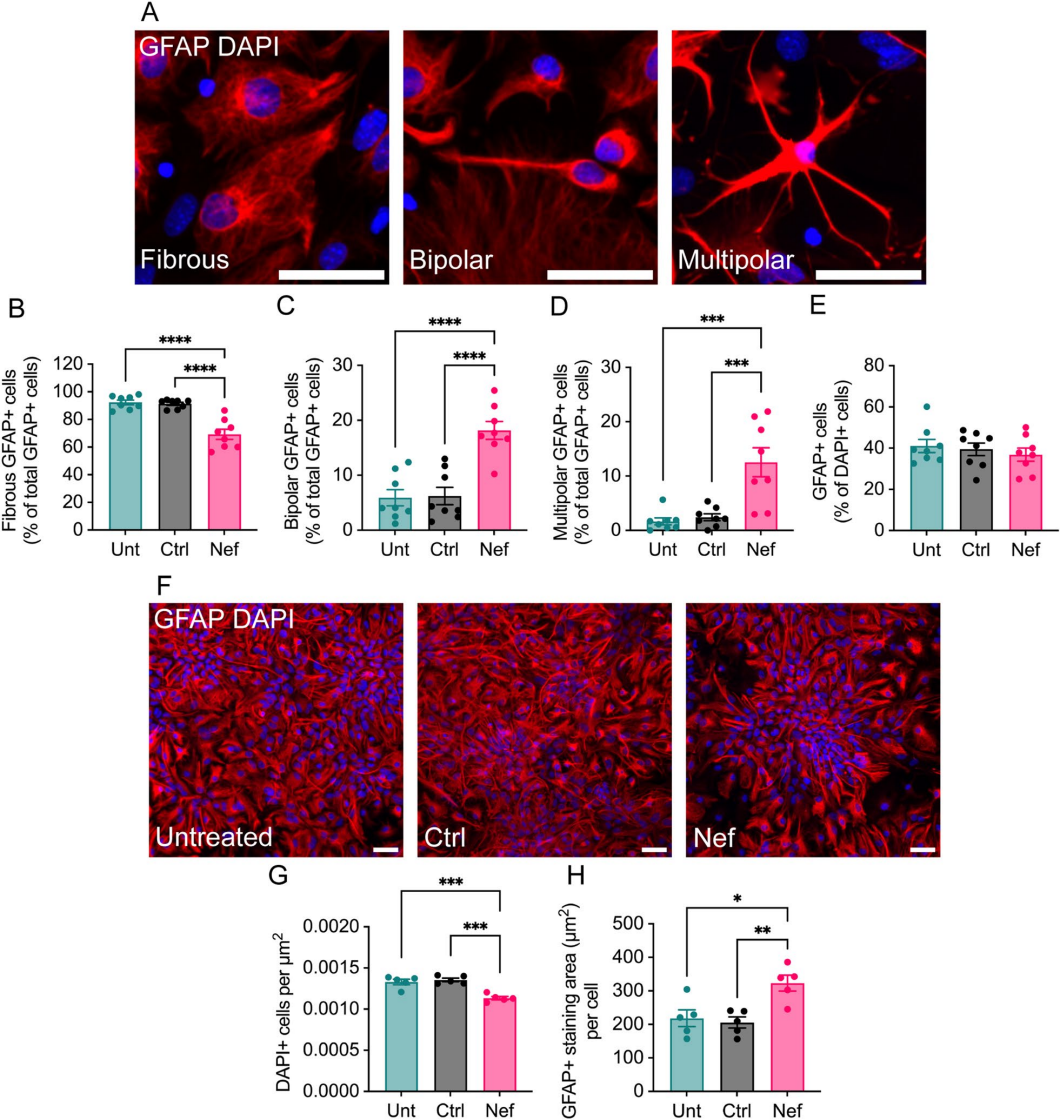
Effects of Nef EVs on astrocyte morphology in vitro. Mouse primary brain cells were collected at postnatal day 3. After 5 days of growth in media to promote oligodendrocyte development, cells were treated with either Ctrl or Nef EVs for 48 h, with untreated (Unt) cultures served as negative controls. **A**. Representative fluorescence images depict the three primary types of astrocyte morphology observed, including (i) fibrous, (ii) bipolar, and (iii) multipolar. GFAP (red) and DAPI (blue) label astrocytes and nuclei, respectively. **B**–**D** Quantification of fibrous, bipolar, and multipolar GFAP + cells as a proportion of the total GFAP + cell population. N = 5 images/group. **E** Quantification of GFAP + cells as a proportion of DAPI + cells. **F** A2B5 + cells were purified via MACS from dissociated mouse brains, cultured for 3 days in media to promote astrocyte differentiation, and then treated with either Ctrl or Nef EVs for 48 h. GFAP (red) and DAPI (blue) label astrocytes and nuclei, respectively. Scale bar is 50 µm. **G** Quantification of DAPI + cells. **H** Quantification of GFAP + cells. N = 5, *p < 0.05; **p < 0.01; ***p < 0.001; ****p < 0.0001

As in vivo (Fig. 2), astrocytes demonstrated a marked shift in morphology in mixed cultures treated with Nef EVs compared to controls (Fig. 6A–D): There was a significant decrease in the proportion of fibrous GFAP + cells (25% decrease relative to untreated, 24% decrease relative to Ctrl EV-treated; Fig. 6B) that was accompanied by significant increase in rod/bipolar (207% relative to untreated, 192% relative to Ctrl EV-treated; Fig. 6C) and multipolar/stellated cells (649% relative to untreated, 415% relative to Ctrl EV-treated; Fig. 6D). There was no change in the number of GFAP + cells across treatment conditions (Fig. 6E), indicating no loss of astrocytes. This shift in astrocyte morphology may reflect phenotypic changes that could impact astrocyte function.

Since Nef EVs had a pronounced effect on astrocytes both in vivo and in mixed cultures and because astrocytes are critical for maintaining oligodendrocyte survival and myelin maintenance [55], we examined the effect of Nef EVs on purified astrocytes (Fig. 6F–H). Treating GFAP + -enriched astrocyte cultures with Nef EVs resulted in a decrease in the total number of DAPI + cells and an increase in the GFAP + staining area per cell (Figs. 6G, H), indicating a decrease in GFAP + astrocytes but increased activation of the remaining astrocytes. Nef EV treatment further resulted in changes in GFAP + cell organization: whereas GFAP + cells in control samples were scattered diffusely with no discernable orientation of astrocyte processes, Nef EV-treated GFAP + cells were aggregated in clusters with processes extending radially around the center point (Fig. 6F). Taken together, these data indicate that Nef EVs directly affect GFAP + astrocytes.

### Nef-induced oligodendrocyte damage is prevented by agents that inhibit the effect of Nef on ABCA1

**Fig. 7:**
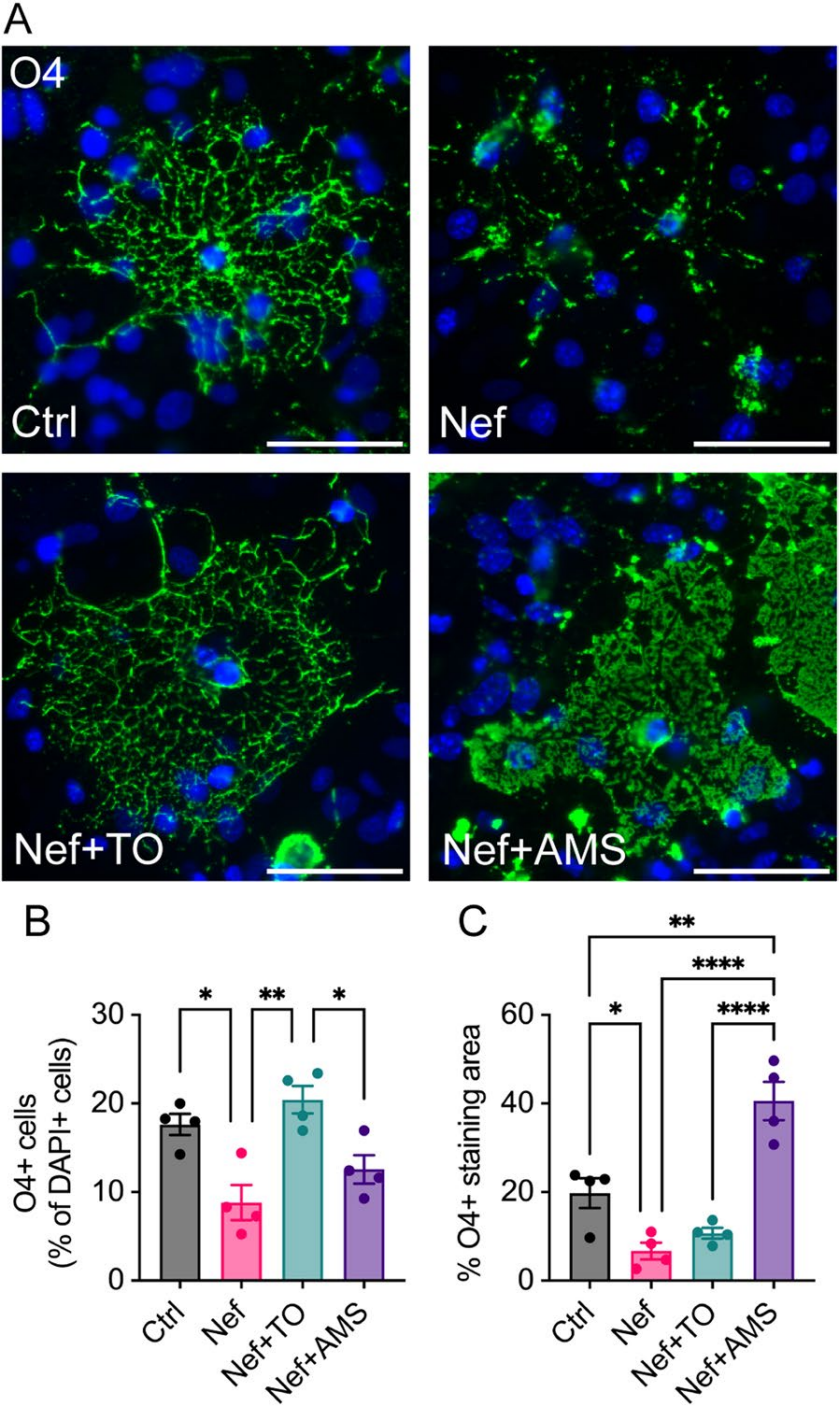
Treatment of brain cells with agents targeting Nef-mediated downmodulation of ABCA1. Mouse primary brain cells were collected at postnatal day 3 and allowed to grow for 7 days in media conducive to oligodendrocyte development. Subsequently, they were pre-treated with the LXR agonist TO-901317 (TO, 1 µM) or the inhibitor of the Nef-calnexin interaction, AMS-55 (AMS, 1 µM), for 3 days, followed by treatment with either Ctrl or Nef EVs for 48 h. **A**—Representative fluorescence images display oligodendrocytes (O4, green) and nuclei (DAPI, blue). **B** Quantification of total O4 + oligodendrocytes as a proportion of total DAPI + cells. **C** Quantification of the percentage of O4 immunofluorescence. Scale bar is 50 µm. N = 4. *p < 0.05; **p < 0.01; ****p < 0.0001

Previously, we demonstrated that Nef EVs decreased ABCA1 protein abundance in peripheral myeloid cells, impairing cholesterol efflux and promoting inflammatory responses [60, 67]. Analysis of ABCA1 in primary mouse brain cultures treated with Nef EVs demonstrated specific downmodulation of ABCA1 (Supplemental Fig. S6). We have shown that the liver X receptor (LXR) agonist TO-901317 restores ABCA1-mediated cholesterol efflux in Nef-expressing HIV-infected macrophages [68, 69]. We also identified the small-molecule compound AMS-55, which potently reversed the negative effects of Nef on ABCA1 abundance by inhibiting the binding of Nef to calnexin, an endoplasmic reticulum chaperone protein integral for ABCA1 maturation [70–72]. To determine the role of Nef EV-mediated disruption of ABCA1 in oligodendrocyte damage, we tested whether this damage could be prevented by treatment with TO-901317 or AMS-55. Mixed brain cultures obtained from neonatal C57BL/6J mice at postnatal day 3 were cultured for 7 days and then pretreated once daily for 3 days with 1 µM TO-901317 or AMS-55. Cultures were then treated once daily with EVs for 2 days before immunostaining for O4. Consistent with the results shown in Fig. 5, treatment with Nef EVs resulted in stark morphological disruption of O4 + cells (Fig. 7A), accompanied by a 50% reduction in total O4 + cell density (Fig. 7B) and a 66% reduction in O4 + cell immunofluorescence (Fig. 7C). These effects of Nef EVs are consistent with our findings in purified oligodendrocyte cultures and support our conclusion that Nef EVs preferentially affect mature oligodendrocytes. Pretreatment with TO-901317 prior to treatment with Nef EVs restored O4 + cell counts to the levels observed in Ctrl EV-treated cultures but only partially restored O4 + immunofluorescence. Conversely, AMS-55 only partially restored O4 + cell density, but O4 + immunofluorescence was more than twice that in Ctrl EV-treated cultures. Taken together, these data suggest that TO-901317 and AMS-55 partially prevent Nef-mediated oligodendrocyte damage and implicate ABCA1 in the mechanism of oligodendrocyte damage caused by Nef EVs.

## Discussion

Previous reports have linked Nef to HAND pathogenesis [33–35, 59], but no studies have investigated the role of Nef in demyelination. In this study, we discovered that Nef-containing EVs impair oligodendrocytes and disrupt CNS myelin. Our findings align with those of Radja et al. who observed a significant reduction in O4 + immunofluorescence and alterations in oligodendrocyte morphology induced by Nef in a transgenic model expressing Nef under the oligodendrocyte-specific MBP promoter [28]. Since HIV-1 does not infect oligodendrocytes due to their lack of CD4 and CCR5 receptors, our study offers a biologically significant context by examining the effects of exogenously produced EV-associated Nef on oligodendrocytes.

Other studies have provided evidence of the toxic effects of both Nef and Nef-containing EVs on neurons [36, 37, 73]. Considering that disruptions in myelin can lead to severe axonal degeneration and impair neuronal function [74], we aimed to investigate the impact of Nef EVs on myelin sheaths. We employed various experimental methods to explore Nef-mediated myelin perturbation and oligodendrocyte damage. Initially, we utilized electron microscopy (EM) analysis after administering Nef EVs in vivo and observed the depletion of myelin sheaths around intact axons. Subsequently, ex vivo treatment of cerebellar slice cultures with Nef EVs revealed an increase in the number of MBP-negative axons, indicating a reduction in axon myelination without significant neuronal death. Finally, in vitro experiments demonstrated that Nef EVs directly inhibited the enrichment of A2B5 + cells in culture, indicating an impact on oligodendrocytes. Taken together, these findings suggest that Nef EVs exert direct effects on myelin and oligodendrocytes, potentially contributing to subsequent neurodegeneration.

Oligodendrocyte maturation is typically classified into four stages based on morphology and the temporal expression of cell surface protein markers (as reviewed in [75, 76]): bipolar oligodendrocyte precursor cells (OPCs), branching premyelinating oligodendrocytes (preOLs), differentiated immature oligodendrocytes, and mature myelinating oligodendrocytes. The sensitivity of oligodendrocytes to programmed cell death varies with maturation: while OPCs express potent mitogens and survival factors, such as PDGF-A [77], preOLs are highly susceptible to apoptosis induced by various stressors [78, 79]. Indeed, the majority of oligodendrocytes are lost during the preOL stage of development and during adult oligodendrogenesis [80–82]. In both mixed primary and enriched A2B5 + oligodendrocyte cultures, we observed more pronounced morphological disruptions in O4 + cells in older cultures. This could be attributed to the heightened susceptibility of oligodendrocytes to stress during the preOL stage, when cells experience increased metabolic demands associated with myelin synthesis pathways.

We demonstrate that agents targeting the effect of Nef on ABCA1 prevent Nef-induced oligodendrocyte damage in vitro. The LXR agonist TO-901317, known for its potent induction of ABCA1, has previously been shown to enhance oligodendrocyte proliferation, differentiation, and maturation, and to alleviate ischemia and lysolecithin-induced demyelination [83, 84]. Although TO-901317 treatment resulted in an overall increase in O4 + cells, we observed two distinct oligodendrocyte morphologies in cultures treated with TO-901317: more mature oligodendrocytes with highly complex branching and less mature oligodendrocytes with fewer branching processes. While TO-901317 prevented O4 + cell loss, its effect on immunofluorescence was less pronounced, suggesting that the ABCA1 pathway might not be the sole target of Nef-mediated oligodendrocyte toxicity. Additionally, the interaction of AMS-55, an inhibitor of the Nef-calnexin complex necessary for Nef-mediated ABCA1 downregulation [70, 72], partially prevented oligodendrocyte damage. AMS-55 did not fully restore the O4 + cell number but did significantly increase the O4 + staining area to a level greater than that observed in Ctrl EV-treated cultures. These findings suggested that while inhibiting the Nef-calnexin interaction (thus restoring ABCA1 abundance) was not sufficient to completely prevent Nef-mediated oligodendrocyte damage, it did protect a certain population of cells. Furthermore, cultures treated with Nef + AMS-55 displayed a sheathing morphology reflective of more mature oligodendrocytes. Overall, these results suggest that oligodendrocyte damage caused by Nef EVs is at least partially mediated by ABCA1. Notably, LXRs are critical regulators of cholesterol homeostasis in the CNS and induce the expression of several genes involved in cholesterol transport in oligodendrocytes [85]. LXRs directly regulate reverse cholesterol transport through the transcription of apoE and cholesterol transporters, including ABCA1, ABCG1, and ABCG4 [86]. LXRs also regulate the uptake of cholesterol via low-density lipoprotein receptors (LDLRs) [87–89]. These findings suggested that the effects of Nef EVs on oligodendrocytes may be due to interactions with several aspects of cholesterol homeostasis regulated by LXRs. Future studies will further examine the effects of Nef EVs on cholesterol metabolism in the CNS.

Microglia and astrocytes are involved in myelin maintenance and repair; alterations to these cells can contribute to demyelination and disrupt the process of remyelination [39], and both cell types have been implicated in various mechanisms of neuronal damage that likely contribute to HAND [90, 91]. Both cell types are likely infected by HIV and are capable of producing Nef [92], but the effect of exogenous Nef on these cells in relation to the subsequent effect on myelin has not been previously reported. Our finding that Nef EVs disrupt oligodendrocytes in enriched cultures implies that Nef-containing EVs directly damage oligodendrocytes in vitro*,* but does not preclude indirect effects from other cell types, including microglia or astrocytes. In fact, our results suggest that in the context of mixed cultures, oligodendrocytes are less susceptible to the cytotoxic effects of Nef EVs. We report that Nef EV injection induces an increase in IBA1 + microglia/myeloid cell infiltration at myelin lesions. However, our ex vivo and in vitro data indicate that systemic inflammation is not essential for Nef EV-mediated disruption of myelin and oligodendrocytes. For example, altered myelination of NF + axons was observed in cerebellar slice cultures, an ex vivo system ideal for studying myelin in the absence of an infiltrating immune response. Additionally, we did not observe changes in cytokine protein expression in mixed primary brain cultures containing oligodendrocytes, astrocytes, and microglia, and observed oligodendrocyte damage in enriched cultures derived from A2B5 + oligodendrocyte precursor cells. While our results show that inflammation is not necessary for oligodendrocyte damage, it likely contributes to the persistent demyelinating pathology observed in individuals with HAND.

Our finding that Nef EVs do not stimulate inflammatory cytokine release by mixed brain cultures appears to contradict the findings of previous reports that Nef exosomes stimulate the release of cytokines and chemokines from CHME-5 microglia [93]. A possible explanation is that IBA1 + microglia/macrophages make up only 10% of the cells in our primary brain cultures, which may be insufficient to elicit a notable inflammatory response. In addition, there may be differences in EV preparation and contamination with proinflammatory agents (e.g., LPS) between studies. Indeed, we have previously shown that Nef alone is unable to elicit a cytokine response in macrophages in vitro, but Nef EVs reorganize lipid rafts and potentiate proinflammatory responses in bystander cells to inflammatory stimuli [60]. This may be particularly relevant to in vivo experimental models, where low-level inflammation is difficult to avoid. Therefore, Nef EV-exposed astrocytes and microglia likely indirectly contribute to oligodendrocyte damage via the potentiation of inflammation*.*

Disruptions to GFAP + astrocytes were observed after Nef EV treatment, including a decrease in cell density and altered co-expression of the astrocytic maturation status and reactivity markers GFAP and vimentin in vivo, and a shift in cell morphology and orientation in vitro. Conflicting reports on the effects of HIV and Nef on astrocytic expression of GFAP have been published: one study reported increased expression of GFAP, indicative of a reactive astrocyte phenotype [94], while the other showed that Nef downregulates GFAP expression in astrocytes in vitro [62]. These conflicting findings may be due to the heterogeneity of CNS astrocytes. In our studies, we consistently observed a disruption in GFAP + cells in Nef EV-injected white matter and in purified astrocyte cultures. This result was unexpected given the astrogliosis commonly observed in the brains of HIV-positive individuals [95]. The principal driver of astrogliosis in HIV-infected brain is likely the viral protein Tat, known to elevate GFAP expression [96]. In the context of HIV brain infection, particularly in the era of ART, the collective effect on astrocytes could vary based on the local concentrations of Tat and Nef, alongside other potential factors. The reduction in GFAP expression observed in our study may signify a specific influence of Nef EVs on astrocytic function, potentially contributing to astrocyte dysfunction during astrogliosis. Suppression of GFAP expression in astrocytes by Nef EVs does not contradict their pro-inflammatory effect and may represent the consequences of abnormal activation. These results suggest a potential shift in phenotype or cellular dysfunction that could affect astrocytic homeostatic mechanisms. Since astrocytes are critical for myelin maintenance [55], Nef EV-mediated astrocyte damage likely contributes to oligodendrocyte damage and myelin impairment.

**Fig. 8:**
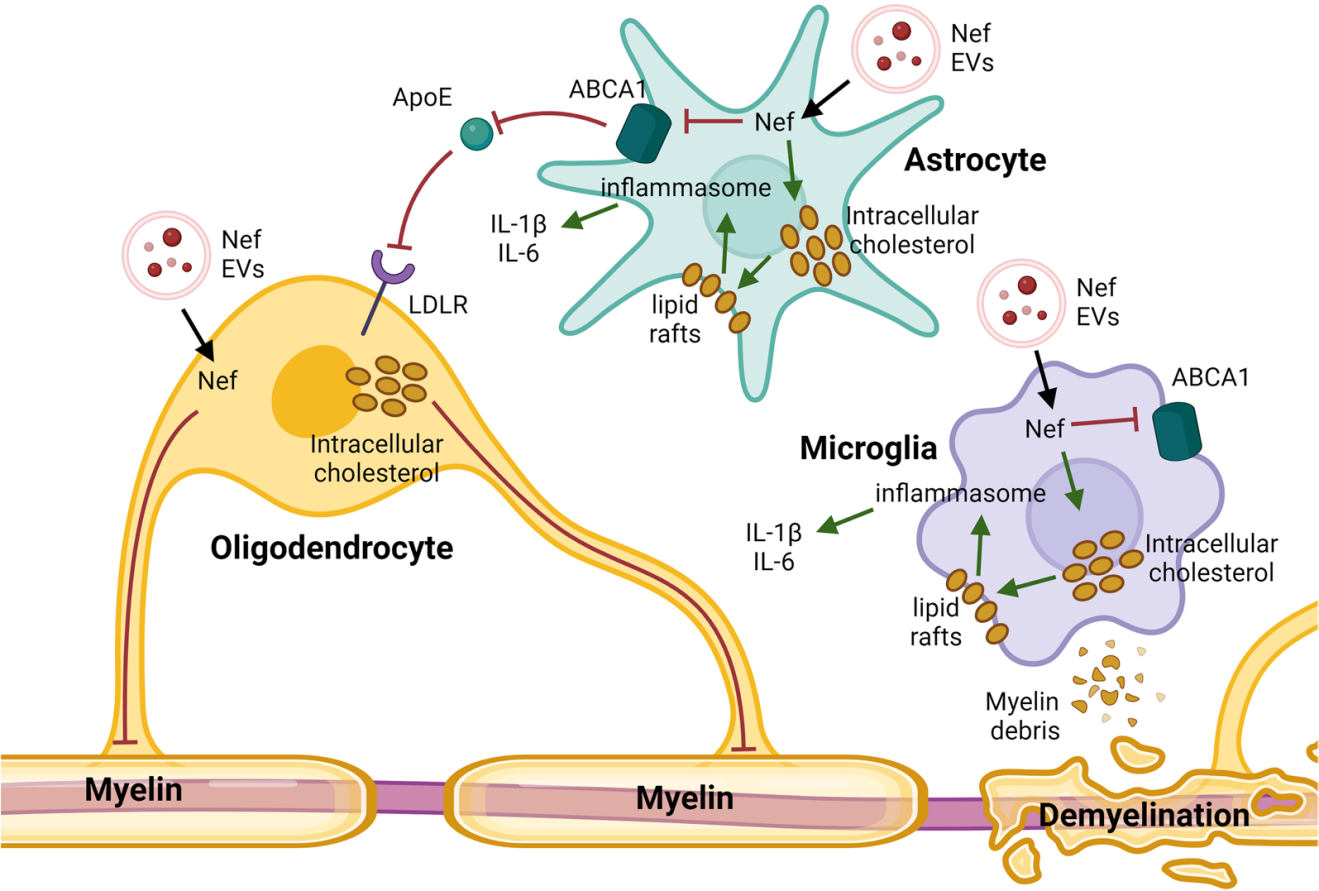
Schematic for Nef EV-mediated oligodendrocyte damage and myelin impairment. Nef is released from extracellular vesicles (EVs) and is internalized by bystander cells in the brain. Nef EVs directly damage oligodendrocytes, compromising their survival or metabolism and impairing myelin integrity. This damage is partially mediated by the downregulation of ABCA1. Additionally, Nef may indirectly harm oligodendrocytes by disrupting ABCA1-mediated cholesterol efflux in astrocytes and microglia. This disruption leads to increased intracellular cholesterol levels, lipid raft formation, activation of inflammasomes, and the secretion of pro-inflammatory cytokines. Inflammation is further exacerbated in microglia during recycling and clearance of myelin debris. In astrocytes, degradation of ABCA1 may hinder the transport of critical cholesterol to oligodendrocytes, further disrupting myelin homeostasis. Image created with BioRender

We propose a mechanism whereby Nef EVs directly and indirectly damage oligodendrocytes and disrupt myelin in the CNS (Fig. 8). Nef EVs are released from HIV-infected cells and taken up by bystander cells in the CNS. Oligodendrocytes are directly damaged by Nef EVs, which compromises the survival and/or metabolism of these cells and impairs myelin integrity. Additionally, Nef EVs may indirectly damage oligodendrocytes by disrupting cholesterol homeostasis in astrocytes and microglia. Nef reduces the amount of ABCA1, the primary cholesterol efflux transporter in these cells [50, 97], through previously described mechanisms [60]. Disrupting ABCA1-mediated cholesterol efflux leads to increased intracellular cholesterol levels, lipid raft formation, inflammasome activation, and proinflammatory cytokine secretion stimulation induced by an inflammatory stimulus, all of which contribute to demyelinating pathology. In astrocytes, degradation of ABCA1 may also prevent the transport of critical cholesterol to oligodendrocytes, which further damages oligodendrocytes and disrupts myelin integrity. The inflammatory process is further exacerbated in phagocytic microglia during the recycling and clearance of cholesterol-rich myelin debris, leading to a persistent inflammatory state.

The relevance of our findings to the pathogenesis of HAND is underscored by the demonstrated expression of Nef and Nef-containing EVs in the brains of HIV-infected individuals, including those undergoing ART treatment [36, 43, 59, 98–100]. Notably, Nef EVs can originate from HIV-infected brain cells such as microglial cells, perivascular macrophages, and astrocytes, which constitute viral reservoirs in the brain. These reservoirs are established shortly after initial infection and persist despite the initiation of ART [101]. Furthermore, circulating Nef EVs [43] represent another potential source of Nef in the brain, as these vesicles possess the capability to traverse the blood–brain barrier [102].

## Limitations

It is important to note that in this study, EVs were derived from HEK293T cells rather than from primary or immortalized CNS cells. Given that EVs facilitate the transfer of proteins, lipids, and nucleic acids (including noncoding RNAs and microRNAs) [103], brain-derived EVs (BDEVs) may contain specific materials that further contribute to Nef-mediated pathology in the CNS. However, the characterization of BDEVs and the methodology for their production are still evolving scientific approaches. In contrast, HEK293T cells are widely recognized as a model system for EV production. In fact, EVs derived from HEK293T cells are extensively characterized and considered reliable, to the extent that they can be commercially purchased for therapeutic delivery (System Biosciences, #EXOP-110A-1) and are commonly utilized in preclinical studies involving EV-based therapeutics to treat both neurological and systemic diseases [104]. Furthermore, our laboratory has extensively characterized the effects of HEK293T-derived Nef EVs on cholesterol metabolism [42, 60, 105], suggesting that these EVs are well-suited for foundational studies of white matter damage caused by Nef EVs. Future studies will aim to investigate the effects of astrocyte- and microglia-derived Nef EVs on myelin and oligodendrocytes in the CNS.

## Conclusions

In conclusion, our study presents the initial characterization of the effects of Nef EVs on myelin loss within the CNS. We observed that Nef EVs induce direct damage to oligodendrocytes and potentially exert indirect effects by perturbing microglia and astrocytes. Demyelination is likely mediated, at least in part, by the impairment of cholesterol homeostasis resulting from Nef EV-mediated downmodulation of ABCA1. Previously reported agents capable of blocking or reversing ABCA1 downregulation by Nef have shown promising results in protecting oligodendrocytes from damage. Further elucidation of the mechanisms underlying Nef EV-mediated oligodendrocyte damage, particularly its impact on cholesterol metabolism, holds potential for identifying therapeutics aimed at preventing or treating HAND. Our study sheds light on the intricate interplay between Nef, glial cells, and myelin integrity, thereby contributing to a deeper understanding of HAND pathogenesis. Additionally, our findings may pave the way for the identification of potential targets for therapeutic intervention aimed at alleviating neurocognitive deficits in individuals living with HIV.

### Supplementary Information

Supplementary Material 1.

## Data Availability

All data generated or analyzed during this study are included in this published article and its supplementary information files.

## Declarations

### Declarations

Ethics approval and consent to participate

All the experimental procedures were approved by the George Washington University Institutional Animal Care and Use Committee (protocol A2020-40, approved on December 15, 2020).
