# Supplementary material for: Extracellular vesicles produced by HIV-1 Nef-expressing cells induce myelin impairment and oligodendrocyte damage in the mouse central nervous system
Source: J Neuroinflammation. 2024 May 13;21:127. doi: 10.1186/s12974-024-03124-5 (PMC11090814; doi:10.1186/s12974-024-03124-5)
Supplement: Supplementary file 1 — Supplementary Material 1. [file 12974_2024_3124_MOESM1_ESM.docx]

**Supplemental Figure S1. Analysis of Nef EVs. A -** EVs produced by HEK293T cells transfected with Nef-expressing (Nef EVs) or empty (Ctrl EVs) vector were analyzed using Tunable Resistive Pulse Sensing technology on the Exoid instrument. Particle concentration was provided by the instrument’s software. **B –** 4.4x10^10^ of Nef EVs were resuspended in 50 μl of ELISA buffer and analyzed by home-made Nef ELISA. Nef concentration was measured at 13.39 ng/ml, corresponding to 0.67 ng of Nef per 4.4x10^10^ EV particles. EVs were kept frozen at -70^o^C for 24 hours prior to analysis.

**
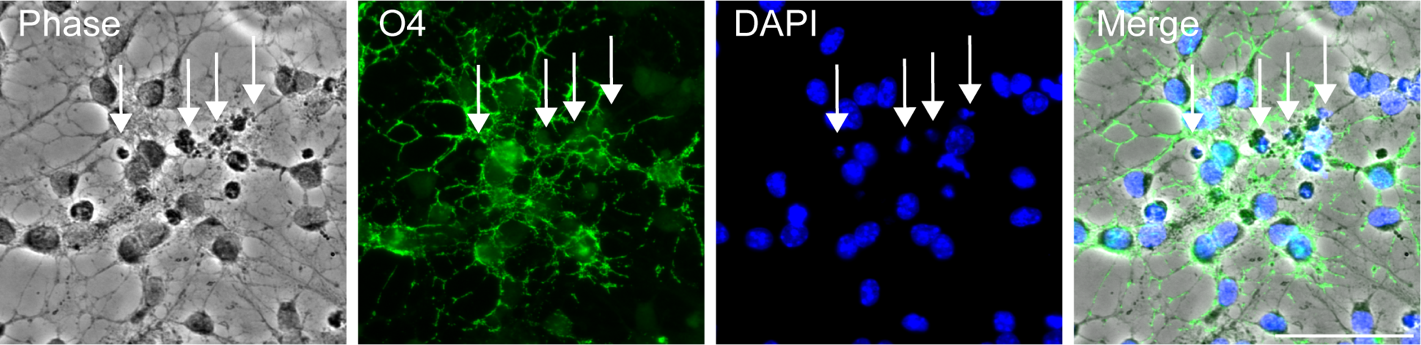
**

**Supplementary Figure S2. Fragmented chromatin overlaps with damaged oligodendrocytes *in vitro*.** A2B5+ cells were purified via magnetic activated cell sorting from dissociated mouse brains at postnatal day 5, grown for 1 d in media to promote oligodendrocyte precursor cell proliferation, then 2 d in media to promote O4+ oligodendrocyte differentiation. Representative phase (gray) and fluorescence images of oligodendrocytes (O4, green) and nuclei (DAPI, blue). DAPI+ chromatin fragments overlay with damaged cells in phase and with disrupted O4+ cells via fluorescence imaging (white arrows). Scale bar is 50 µm.


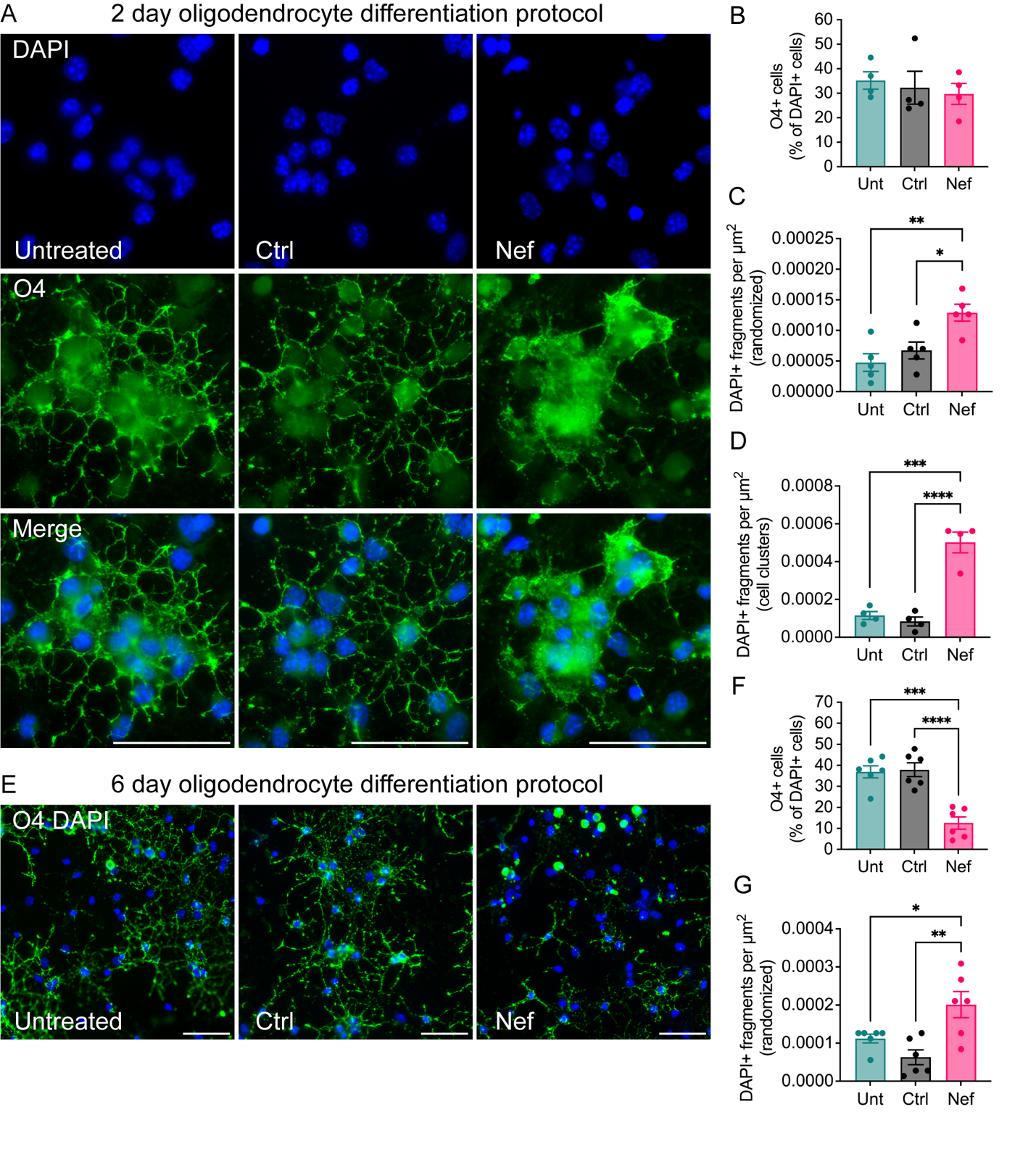


**Supplementary Figure S3. Nef EVs disrupt enriched oligodendrocytes *in vitro*.** A2B5+ cells were purified via magnetic activated cell sorting from dissociated mouse brains at postnatal day 5, grown for 1 d in media to promote oligodendrocyte precursor cell proliferation, then 2 or 6 d in media to promote O4+ oligodendrocyte differentiation, then treated with Ctrl or Nef EVs for 48 h; untreated (Unt) cultures served as negative controls. **A-D.** 2 d oligodendrocyte differentiation protocol. **A.** Representative fluorescence images of oligodendrocytes (O4, green) and nuclei (DAPI, blue). **B.** Quantification of O4+ oligodendrocytes per field. **C-D.** Quantification of DAPI+ chromatin fragments (≤5 µm) per µm^2^ in randomized fields (**C**) and in cell clusters (**D**). **F-G.** 6 d oligodendrocyte differentiation protocol. **E.** Representative fluorescence images of oligodendrocytes (O4, green) and nuclei (DAPI, blue). **F.** Quantification of % O4+ oligodendrocytes as a proportion of DAPI+ cells. **G.** Quantification of DAPI+ chromatin fragments (≤5 µm) per µm^2^ in randomized fields. Scale bar is 50 µm. **P* < 0.05; ***P* < 0.01; ****P* < 0.001; *****P*<0.0001.


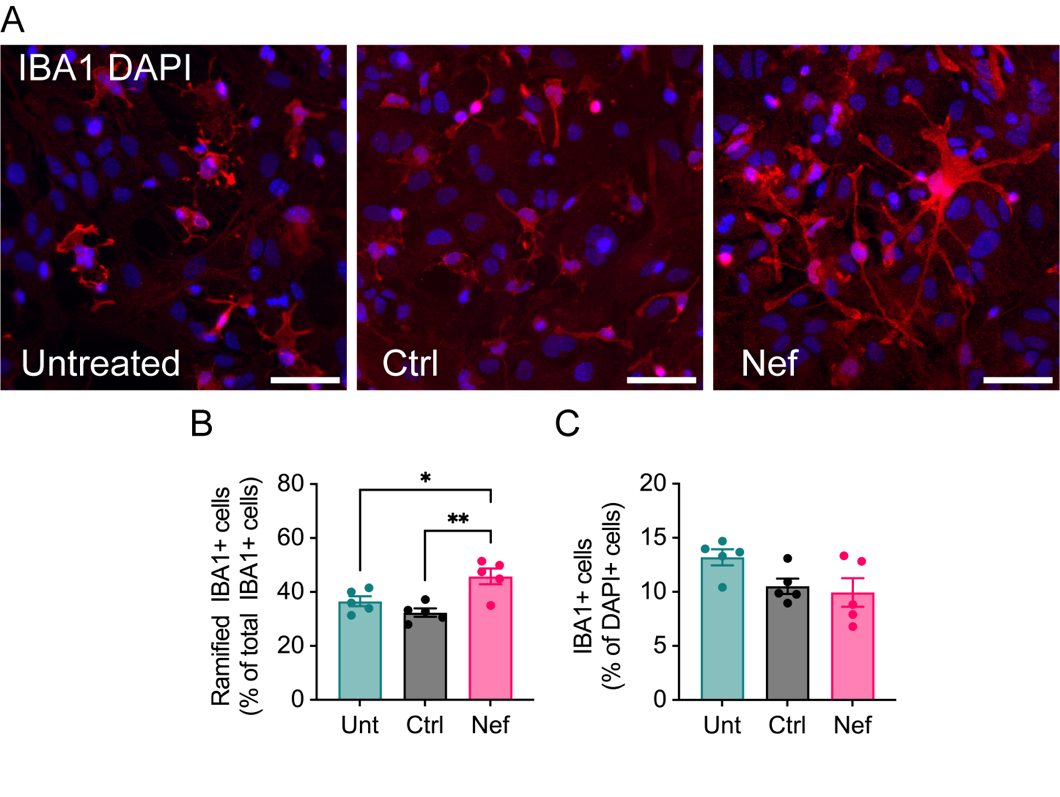


**Supplementary Figure S4. Nef EVs alter microglia morphology in primary brain cultures.** Mouse primary brain cells were cultured at postnatal day 3, grown for 5 days in media to promote oligodendrocyte growth, then treated with Ctrl or Nef EVs for 48 h; untreated (Unt) cultures served as negative controls. **A.** Representative fluorescence images of microglia (IBA1, red) and nuclei (DAPI, blue) 48 h after treatment with Nef EVs. **B.** Quantification of ramified IBA1+ cells as a proportion of total IBA1+ cells 48 h after treatment with Nef EVs. **C.** Quantification of IBA1+ cells as a proportion of DAPI+ cells 48 h after treatment with Nef EVs shows no significant difference between treatment conditions. Scale bar is 50 µm. **P* < 0.05; ***P* < 0.01.


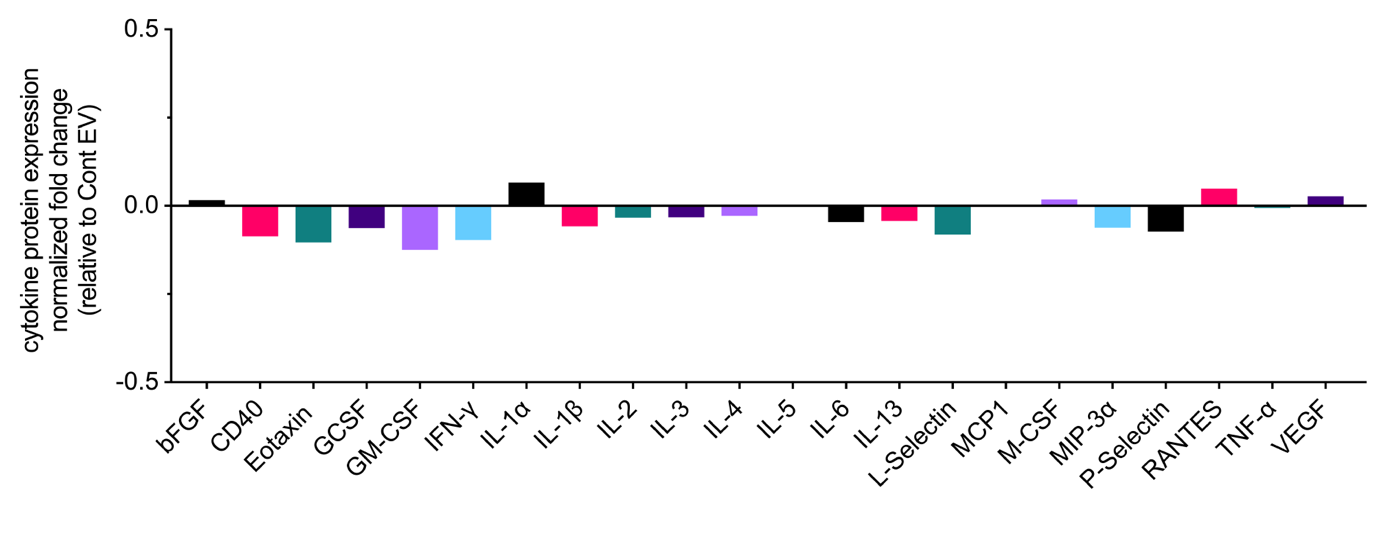


**Supplementary Figure S5. Nef EVs do not alter pro-inflammatory cytokine expression in primary brain cultures.** Mouse primary brain cells were cultured at postnatal day 3, grown for 5 days in media to promote oligodendrocyte growth, then treated with Ctrl or Nef EVs for 48 h. Cytokine levels were quantified using mouse inflammation cytokine array for multiplex protein detection. Raw intensity data was normalized to positive control of each membrane, and fold change was calculated to Ctrl EV values.

**Supplementary Figure S6. Analysis of ABCA1 in primary mixed brain cultures.** Mouse primary brain cells were cultured at postnatal day 3 and grown for 5 days in media to promote oligodendrocyte growth, then treated with Ctrl or Nef EVs for 48 h; untreated (No treatment) cultures served as negative controls. ABCA1 and GAPDH were analyzed on Jesse BioTechne automated western blot instrument (**A**), and results were quantified using BioTechne Compass software (**B**).
